# Exploitation of Nanoparticle–Essential Oil Combinations to Enhance the Efficacy of Antimicrobial Agents Against *Staphylococcus equorum*

**DOI:** 10.3390/pharmaceutics18081017

**Published:** 2026-08-17

**Authors:** Simona Hisirová, Patrícia Hudecová, Vanda Hajdučková, Stanislav Lauko, Nikola Dančová, Lívia Mačák, Oksana Velgosova, Peter Paľove-Balang, Ján Király

**Affiliations:** 1 Department of Microbiology and Immunology, The University of Veterinary Medicine and Pharmacy in Košice, 041 81 Košice, Slovakia; simona.hisirova@student.uvlf.sk (S.H.); patricia.hudecova@student.uvlf.sk (P.H.); vanda.hajduckova@uvlf.sk (V.H.); stanislav.lauko@uvlf.sk (S.L.); 2 Department of Public Veterinary Medicine and Animal Welfare, The University of Veterinary Medicine and Pharmacy in Košice, 041 81 Košice, Slovakia; nikola.dancova@uvlf.sk; 3 Faculty of Mechanical Engineering, Technical University of Košice, Letná 9, 042 00 Košice, Slovakia; livia.macak@tuke.sk (L.M.); oksana.velgosova@tuke.sk (O.V.); 4 Institute of Biology and Ecology, Faculty of Science, Pavol Jozef Šafárik University in Košice, 041 54 Košice, Slovakia; peter.palove-balang@upjs.sk

**Keywords:** nanoparticles, silver, green synthesis, essential oils, *Salvia rosmarinus*, *Lavandula angustifolia*, ampicillin, multidrug resistance, biofilm, *Staphylococcus equorum*

## Abstract

**Background:** This study evaluated the antibacterial, antibiofilm, and biofilm eradication activities of biogenically synthesized silver nanoparticles (AgNPs-L, AgNPs-R) mediated by extracts of *Lavandula angustifolia* and *Salvia rosmarinus*, individually and in combination with their respective essential oils (EOs) and ampicillin (AMP), against a multidrug-resistant biofilm-forming *Staphylococcus equorum* strain. Physicochemical characterization confirmed the successful biosynthesis of spherical AgNPs-L (10–25 nm) and AgNPs-R (5–15 nm). Individual treatments exhibited distinct antibacterial activity, with MIC values of 25 µg/mL for AgNPs-L, 12.5 µg/mL for AgNPs-R, and 0.1% (*v*/*v*) for both EOs; however, they showed no ability to eradicate preformed biofilms. Dual AgNPs/EO combinations at subinhibitory concentrations demonstrated borderline additive effects (FICI = 0.501) and significantly potentiated antibacterial and antibiofilm activity compared with individual treatments. Triple AgNPs/EO/AMP combinations exhibited the most pronounced biological effects, with predominantly additive interactions depending on AMP concentration. Lavender-based triple combinations achieved up to 63.9% inhibition of planktonic growth, 78.1% prevention of biofilm formation, and 37.3% eradication of mature biofilms, whereas rosemary-based combinations resulted in 57.1%, 69.4%, and 42.7% inhibition, respectively. These findings highlight the potential of multi-component systems integrating biogenic nanomaterials, phytochemicals, and conventional antibiotics as a promising strategy to enhance antimicrobial efficacy against persistent biofilm-forming non-aureus staphylococci.

## 1. Introduction

Bacterial adaptation to adverse environmental conditions, including exposure to antimicrobial agents, plays a pivotal role in the selection and dissemination of multidrug-resistant (MDR) strains [1]. According to the World Health Organization (WHO), antimicrobial resistance (AMR) is one of the most pressing challenges facing modern medicine and public health, requiring coordinated, multidisciplinary efforts at the global level to mitigate its impact [2]. In addition to the inappropriate use of antibiotics, the growing burden of AMR is further exacerbated by the complexity of bacterial adaptation, particularly the horizontal transfer of genetic resistance determinants, which accelerates the emergence and dissemination of resistant strains [3]. Another critical factor contributing to bacterial persistence is biofilm formation, which enhances bacterial survival within the host while markedly reducing the effectiveness of both host immune defenses and antimicrobial therapy, thereby promoting persistent infections and compromising therapeutic outcomes [4]. Consequently, alongside the optimization of antibiotic stewardship, increasing attention has been directed toward the development of alternative antimicrobial strategies and novel therapeutic approaches to combat resistant bacterial infections [5].

Staphylococci significantly contribute to the global spread of AMR due to their high genetic plasticity and ability to form biofilms [6]. While research has traditionally focused on *Staphylococcus aureus*, increasing attention has been directed toward non-aureus staphylococci and mammaliicocci (NASM), which represent important reservoirs of antimicrobial resistance genes with potential for horizontal transfer [7,8]. Among NASM species, *Staphylococcus equorum* has emerged as a relevant model for evaluating novel antimicrobial strategies. This species is commonly found in veterinary and food-production environments, but it may also harbor resistance determinants against clinically relevant antibiotics, including penicillins, macrolides, and tetracyclines [8,9]. Its ability to form biofilms further increases its relevance, as biofilm-associated cells exhibit enhanced tolerance to antimicrobial agents and disinfectants [10].

Silver nanoparticles (AgNPs) represent one of the most extensively studied nanomaterials for antimicrobial applications due to their strong antibacterial and antibiofilm properties and broad biomedical potential [11]. Recently, green synthesis approaches using plant extracts, microorganisms, or their metabolites have gained considerable attention as sustainable alternatives to conventional chemical synthesis [12]. Plant-derived metabolites can act as reducing and capping agents, influencing the physicochemical properties and biological activity of the resulting nanoparticles [13]. Biosynthesized AgNPs may therefore exhibit enhanced antimicrobial effects compared with chemically synthesized nanoparticles, partly due to the synergistic contribution of surface-associated phytochemicals [14]. Their small size and high surface-to-volume ratio facilitate interactions with bacterial cell envelopes, promoting nanoparticle internalization and activation of multiple mechanisms involved in bacterial growth inhibition and biofilm disruption [15].

Plant-derived essential oils (EOs) represent another promising group of natural antimicrobial agents and are increasingly investigated as eco-friendly alternatives to conventional antibiotics [16]. Lavender (*Lavandula angustifolia*) and rosemary (*Salvia rosmarinus*) are important sources of bioactive EOs rich in terpenoids, phenolic compounds, and other secondary metabolites. These complex mixtures exhibit antibacterial and antibiofilm activities through multiple mechanisms, including disruption of bacterial membrane integrity and modulation of biofilm-associated processes [17,18]. However, their therapeutic application is limited by high volatility, poor solubility, and low stability, which may be improved through combination with nanoparticle-based systems to enhance their antimicrobial efficacy [19,20].

Therefore, this study aimed to evaluate and compare the antibacterial, antibiofilm, and biofilm eradication activities of biosynthesized AgNPs derived from lavender (*Lavandula angustifolia*) and rosemary (*Salvia rosmarinus*), their essential oils, and their combinations with AMP. Furthermore, the potentiating effects of these combinations at subinhibitory concentrations were assessed as a potential adjunctive approach to antimicrobial therapy.

## 2. Materials and Methods

### 2.1. Selection of Bacterial Strains for Testing

For the evaluation of the antibacterial, antibiofilm, and biofilm eradication activities of the tested biosynthesized AgNPs derived from lavender and rosemary, their EOs, and their combinations, the strain *Staphylococcus equorum* SD75 was selected. This strain belongs to the collection of the Department of Microbiology and Immunology, University of Veterinary Medicine and Pharmacy in Košice. It represents a clinical staphylococcal isolate classified within the group of NASM, isolated from canine skin. In previous studies, the strain was characterized by both phenotypic and genotypic methods, focusing on selected characteristics, including multidrug resistance, the presence of the methicillin resistance-associated gene *mecA*, efflux pump activity, and strong biofilm-forming ability. The strain was sequenced based on the 16S rRNA gene and deposited in the GenBank database under accession number PV688033. Its characteristics have been described in the study by Hisirová et al. [21]. Detailed characteristics of the strain are presented in Table 1.

### 2.2. Materials

Silver nitrate (AgNO_3_, purity > 98%, Mikrochem Ltd., Pezinok, Slovakia) was used as the silver precursor for nanoparticle synthesis. Dried leaves of *Lavandula angustifolia* (lavender) and *Salvia rosmarinus* (rosemary) were used as plant materials for the preparation of extracts employed in the green synthesis of silver nanoparticles.

Both plant sources were selected due to their rich content of bioactive phytochemicals, including phenolic compounds, flavonoids, and terpenoids, which can act as reducing as well as stabilizing agents during nanoparticle formation [22,23,24].

All solutions were prepared using distilled water.

### 2.3. Preparation of Lavender and Rosemary Extract

Dried leaves of *Lavandula angustifolia* (lavender) and *Salvia rosmarinus* (rosemary) were used for the preparation of plant extracts. Freshly harvested plants were air-dried naturally on the stems at room temperature for 3 days. After drying, the leaves were manually separated from the stems. Prior to extraction, the dried leaves were washed twice with deionized water to remove surface impurities and dust particles. The leaves were subsequently mechanically crushed using a mortar to obtain small fragments and facilitate the release of bioactive compounds.

For each extract, 2.5 g of the crushed dried leaves was mixed with 100 mL of distilled water. The mixtures were heated at approximately 80 °C for 10 min under constant stirring at 600 rpm. After cooling to room temperature, the extracts were filtered through filter paper to remove coarse solid residues and subsequently centrifuged at 9000 rpm for 10 min. Following centrifugation, insoluble impurities formed a sediment at the bottom of the centrifuge tube, while only the clear supernatant was collected and used for the biosynthesis of silver nanoparticles. The freshly prepared extracts were used immediately for nanoparticle synthesis, and aliquots were collected for Fourier-transform infrared spectroscopy (FTIR) analysis. The RP-HPLC-DAD phytochemical profiles of the prepared extracts are provided in the Supplementary Materials (Tables S10 and S11 and Figures S6 and S7).

### 2.4. Preparation of AgNPs Colloids

Silver nanoparticle colloids were prepared using a green synthesis approach with plant extracts derived from *Lavandula angustifolia* and *Salvia rosmarinus*. A precursor solution containing Ag^+^ ions at a concentration of 500 mg/L was prepared from AgNO_3_ using distilled water. The solution was heated to ~80 °C under constant stirring at 600 rpm.

After reaching the desired temperature, the respective plant extract was added to the precursor solution in a precursor-to-extract volume ratio of 10:2. The synthesis was carried out separately for each extract under identical experimental conditions to ensure reproducibility and enable direct comparison of their reducing and stabilizing efficiencies.

Following the addition of the extract, the reaction mixtures were maintained at approximately 70 °C for 30 min under continuous stirring. The formation of silver nanoparticles was preliminarily indicated by a visible color change in the reaction mixture from light yellow to dark brown, which is attributed to the excitation of localized surface plasmon resonance (LSPR), a characteristic optical property of metallic silver nanoparticles [7,12,13]. The resulting colloidal solutions were allowed to cool to room temperature and were subsequently used for physicochemical characterization and biological analyses, through which the successful synthesis of AgNPs was confirmed.

### 2.5. Characterization Methods

Fourier-transform infrared spectroscopy (FTIR) was used to identify the functional groups present in the plant extracts and their possible involvement in the reduction and stabilization of AgNPs. The spectra were recorded using a Bruker Tensor 27 spectrometer (Bruker, Billerica, MA, USA) in the spectral range of 4000–400 cm^−1^ at room temperature. Freshly prepared aqueous plant extracts and AgNP colloidal solutions were dried at 45 °C, mixed with spectroscopic-grade KBr, and compressed into pellets prior to analysis. A pure KBr pellet was used as the background reference.

The formation and stability of silver nanoparticles were monitored using ultraviolet–visible spectrophotometry (UV–Vis; UNICAM UV4, Unicam Ltd., Cambridge, UK) in the wavelength range of 300–700 nm. Measurements were performed at room temperature using quartz semi-micro cuvettes with an optical path length of 10 mm. The appearance of the characteristic surface plasmon resonance (SPR) band confirmed the formation of AgNPs.

The morphology and particle size of the synthesized nanoparticles were analyzed using transmission electron microscopy (TEM; JEOL JEM-2000FX, JEOL Ltd., Tokyo, Japan) operated at an accelerating voltage of 200 kV. For TEM analysis, a drop of the colloidal suspension was deposited onto a copper grid coated with a carbon film, allowed to dry at room temperature, and subsequently coated with an additional carbon layer to improve sample stability. Particle size distribution was evaluated from TEM micrographs using ImageJ software 1.53e (National Institutes of Health and the Laboratory for Optical and Computational Instrumentation (LOCI, University of Wisconsin, Madison, WI, USA)) based on multiple independent images.

Surface morphology and elemental composition were further investigated by scanning electron microscopy coupled with energy-dispersive X-ray spectroscopy (SEM–EDS; ZEISS AURIGA Compact, Carl Zeiss, Oberkochen, Germany).

The hydrodynamic particle size, polydispersity index (PDI), and ζ-potential were determined using a Zetasizer Nano ZS (Malvern Instruments Ltd., Malvern, UK) equipped with a 4 mW He–Ne laser (633 nm). Particle size measurements were performed at a backscatter detection angle of 173°, while ζ-potential was measured using DTS1070 disposable capillary cells. Each sample was measured repeatedly to ensure the reproducibility of the obtained results.

Overall, the synthesis mechanism reflects a complex interplay between reduction kinetics and surface stabilization, which determines the final size, morphology, and stability of the nanoparticles.

### 2.6. Essential Oils

Lavender essential oil (LEO) was extracted from *Lavandula angustifolia* L., whereas rosemary essential oil (REO) was obtained from *Salvia rosmarinus* Spenn. (syn. *Rosmarinus officinalis* L.). Both essential oils were supplied by Calendula a.s. (Nová Ľubovňa, Slovakia), which also performed the analysis of their chemical composition using gas chromatography–mass spectrometry (GC/MS). The relative abundances of the major identified constituents are presented in Table 2 and Table 3.

### 2.7. Bacterial Strain

An 18 h culture of *S. equorum* grown on blood agar at 37 °C was used to evaluate the antimicrobial activity of silver nanoparticles and essential oils. Following incubation, bacterial colonies were resuspended in sterile physiological saline to obtain a suspension corresponding to a 1.0 McFarland standard.

### 2.8. Antibacterial Activity of Nanoparticles, Essential Oils, and Combinations of Nanoparticles and Essential Oils with Ampicillin

The antibacterial activity of the tested samples was quantitatively evaluated by determining the minimum inhibitory concentration (MIC) using the broth microdilution method in sterile 96-well microtiter plates (Brand, Wertheim, Germany) according to the standardized procedures of the European Committee on Antimicrobial Susceptibility Testing (EUCAST) and the Clinical and Laboratory Standards Institute (CLSI). The antibacterial activity of AgNPs and EOs was assessed using two-fold serial dilutions prepared in modified Brain Heart Infusion Broth (mBHI; HiMedia Laboratories, Mumbai, India) supplemented with 1% glucose and 2% sodium chloride (NaCl). A stock solution of AgNPs at a concentration of 500 µg/mL was used. Due to their hydrophobic nature, the essential oils were initially diluted from the 100% stock solution to 10% (*v*/*v*) in polysorbate 20 (AppliChem, Darmstadt, Germany), followed by further dilution in mBHI. Ampicillin (AMP; Sigma-Aldrich, St. Louis, MO, USA) was diluted to subinhibitory concentrations from a stock solution of 10 mg/mL. The tested concentration ranges were 6.25–100 µg/mL for AgNPs, 0.0001–1% (*v*/*v*) for LEO and REO, and 0.25 and 0.5 µg/mL for AMP.

Wells containing the tested samples consisted of 100 µL of bacterial suspension adjusted to a final concentration of 0.5 McFarland and 100 µL of the tested compound at the corresponding final concentration. Background controls contained 100 µL of sterile physiological saline and 100 µL of the tested sample diluted in mBHI at the respective concentration. The positive growth control contained 100 µL of bacterial suspension in physiological saline at a final concentration of 0.5 McFarland and 100 µL of mBHI. Negative controls (sterility controls) contained 100 µL of physiological saline and 100 µL of mBHI. The concentrations of the tested samples were prepared to reach their final values after two-fold dilution with the bacterial suspension. Each concentration was tested in triplicate.

The microtiter plates were incubated aerobically for 24 h at 37 °C with continuous shaking. After incubation, bacterial growth inhibition was determined by measuring absorbance at 600 nm using a Synergy 4 microplate reader (Merck, Darmstadt, Germany). The MIC was defined as the lowest concentration of the tested compound that completely inhibited visible microbial growth.

### 2.9. Antibiofilm Activity of Silver Nanoparticles, Essential Oils, and Combinations of Nanoparticles and Essential Oils with Ampicillin

The antibiofilm activity of the tested samples was evaluated using the microtiter plate method in sterile 96-well plates (Brand, Wertheim, Germany). The preparation of the tested samples, including dilution procedures, applied concentrations, and controls, was performed according to the protocol described in Section 2.8. Each experiment was conducted in three independent replicates. The microtiter plates were incubated aerobically at 37 °C for 24 h under continuous shaking. Following incubation, biofilm formation was determined using a modified crystal violet staining assay based on the method described by O’Toole [25], while inhibition of biofilm formation was quantified spectrophotometrically by measuring absorbance at 550 nm using a Synergy 4 microplate reader (Merck, Darmstadt, Germany).

### 2.10. Biofilm-Eradicating Effect of Nanoparticles, Essential Oils, and Nanoparticles with Ampicillin

The biofilm eradication activity of the tested samples was evaluated using a microtiter plate assay on a preformed biofilm. The biofilm of *S. equorum* was established by incubating bacterial cells in mBHI in microtiter plate wells for 24 h. After removal of planktonic cells by aspiration, the tested samples (100 µL) at selected concentrations were added to the wells together with 100 µL of mBHI. Background correction was performed using wells containing 100 µL of the tested samples at the respective concentrations and 100 µL of physiological saline. Wells containing preformed biofilm supplemented with 200 µL of mBHI served as positive controls, whereas wells containing 200 µL of sterile mBHI served as negative controls (sterility controls). The concentrations of the tested samples corresponded to those used for the evaluation of antibacterial and antibiofilm activities. Each experiment was performed in three independent replicates.

The microtiter plates were subsequently incubated aerobically for 24 h at 37 °C under continuous shaking. Following incubation, the residual adherent biofilm biomass was determined using a modified crystal violet staining assay based on the method described by O’Toole [25]. The amount of biofilm biomass was quantified spectrophotometrically by measuring absorbance at 550 nm using a Synergy 4 microplate reader (Merck, Darmstadt, Germany).

### 2.11. Calculation of the Inhibition Percentage

The percentage of bacterial growth inhibition was determined by comparing bacterial growth in wells containing the AgNPs/EO/AMP combinations with the growth observed after exposure to the individual components alone. Bacterial growth was quantified by measuring absorbance at 600 nm using the method described in Section 2.8. The percentage of inhibition was calculated according to the following equation:

$$\%\;inhibition=\;\frac{A\;single\;-\;A\;combination}{A\;single}\;\times 100$$

where A_combination_ represents the absorbance value of the AgNPs/EO/AMP combination, and A_single_ represents the absorbance value of the individual component.

### 2.12. Calculation of the Fractional Inhibitory Concentration Index (FICI)

The synergistic antibacterial effect of the combination was quantified using the fractional inhibitory concentration index (FICI). Since a fixed sub-MIC (subinhibitory MIC) combination design was applied, the effect of the combination was evaluated in relation to the MIC values of the individual components determined separately. The fractional inhibitory concentration (FIC) for each compound was calculated according to the following equation:

$$FIC\;(AgNPs,\;EO,\;AMP)=\;\frac{MIC\;combination}{MIC\;\left(AgNPs,\;EO,\;AMP\right)\;single}\;$$

where MIC_combination_ represents the MIC value of the AgNPs/EO/AMP combination, and MIC (AgNPs, EO, AMP)_single_ represents the MIC value of the individual component tested separately.

The FIC index was calculated according to the following equation:

$$FICI=\;FIC\;\left(AgNPs\right)+FIC\;\left(EO\right)+FIC\;(AMP)$$

where FIC (AgNPs, EO, AMP) represents the FIC value of each individual component.

The interaction was interpreted as synergistic (FICI ≤ 0.5), additive (0.5 < FICI ≤ 1), indifferent (1 < FICI ≤ 4), or antagonistic (FICI > 4) according to previously described criteria [26]. The percentage of inhibition and FICI values were evaluated exclusively for the AgNPs/EO/AMP combination, as the primary objective of the study was to demonstrate its antibacterial efficacy and potential synergistic effect.

### 2.13. Statistical Analysis

The antibacterial, antibiofilm, and biofilm eradication activities were statistically analyzed using one-way ANOVA followed by Dunnett’s test in GraphPad Prism software version 8.3.0 (GraphPad Software, San Diego, CA, USA). All experiments were performed in three independent replicates, and the results for each isolate are presented as mean values ± standard deviation (SD).

## 3. Results

In this study, the antibacterial, antibiofilm, and biofilm eradication activities of the tested AgNPs, essential oils, and their combinations with AMP were investigated. The obtained results were compared to determine their effects on bacterial growth inhibition and biofilm-associated properties.

### 3.1. AgNPs Biosynthesis and Characterization

Silver nanoparticles (AgNPs) were successfully synthesized using green synthesis approaches based on *Lavandula angustifolia* and *Salvia rosmarinus* extracts. The formation of AgNPs was initially indicated by a visible color change in the reaction mixtures from light yellow to dark brown, suggesting the reduction of Ag^+^ ions and nanoparticle formation.

#### 3.1.1. Optical Properties (UV-Vis)

**Figure 1:**
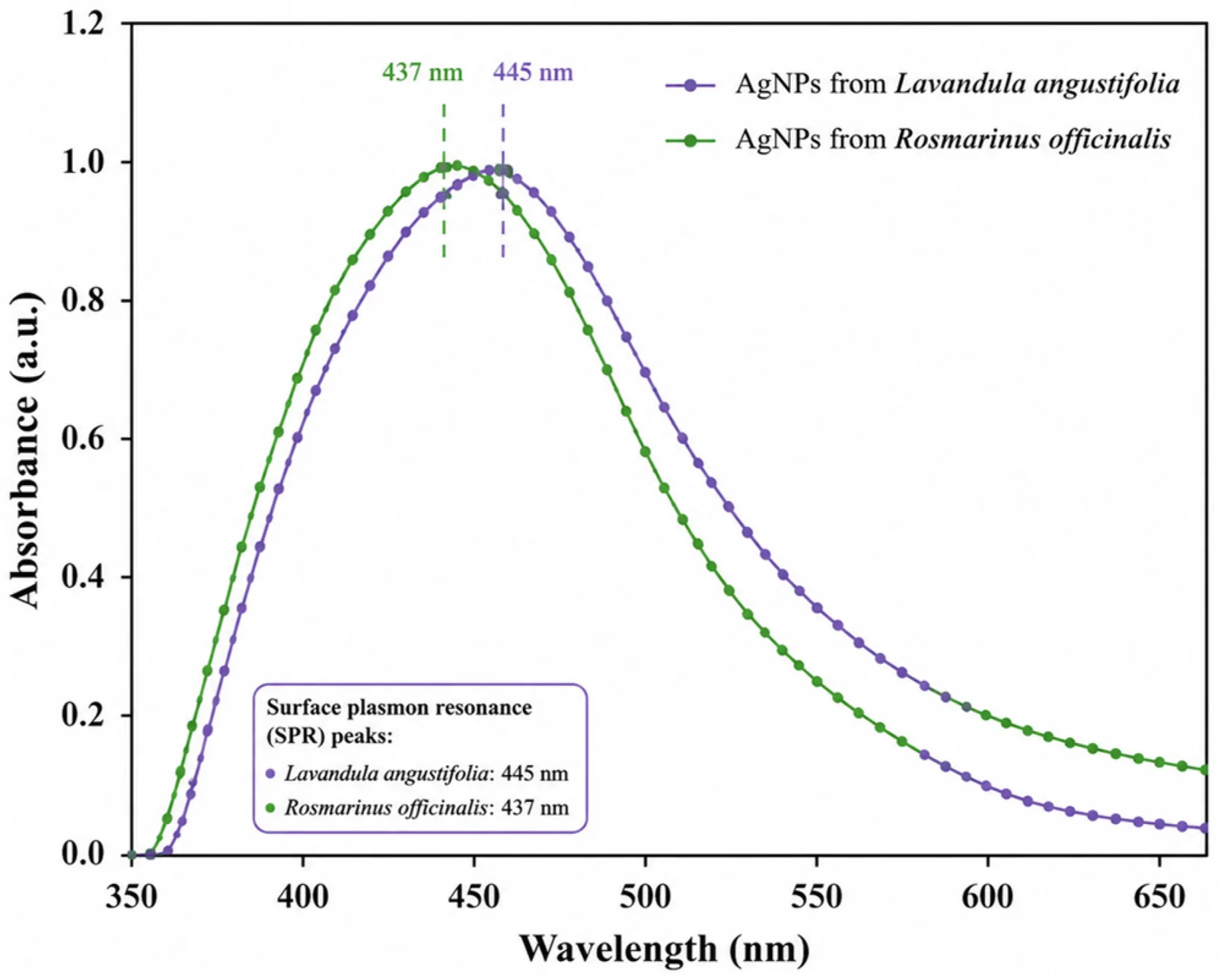
UV–Vis absorption spectrum of AgNPs synthesized using extracts.

The formation of silver nanoparticles was confirmed by UV–Vis spectroscopy through the appearance of characteristic SPR bands [27,28] (Figure 1). Both samples exhibit a distinct absorption peak in the visible region, corresponding to the collective oscillation of conduction electrons on the nanoparticle surface.

The AgNPs synthesized using *Lavandula angustifolia* extract show an SPR maximum at 445 nm, while those prepared using *Salvia rosmarinus* extract exhibit a slightly blue-shifted peak at 437 nm [29]. This shift indicates differences in particle size and distribution, with the rosemary-derived nanoparticles tending toward smaller dimensions.

The relatively broad absorption bands observed for both samples suggest a moderate polydispersity, which is in agreement with the TEM and dynamic light scattering (DLS) analyses. Additionally, the absence of secondary peaks in the spectra indicates the formation of predominantly spherical nanoparticles without significant aggregation.

#### 3.1.2. Morphology and Size Distribution (TEM)

**Figure 2:**
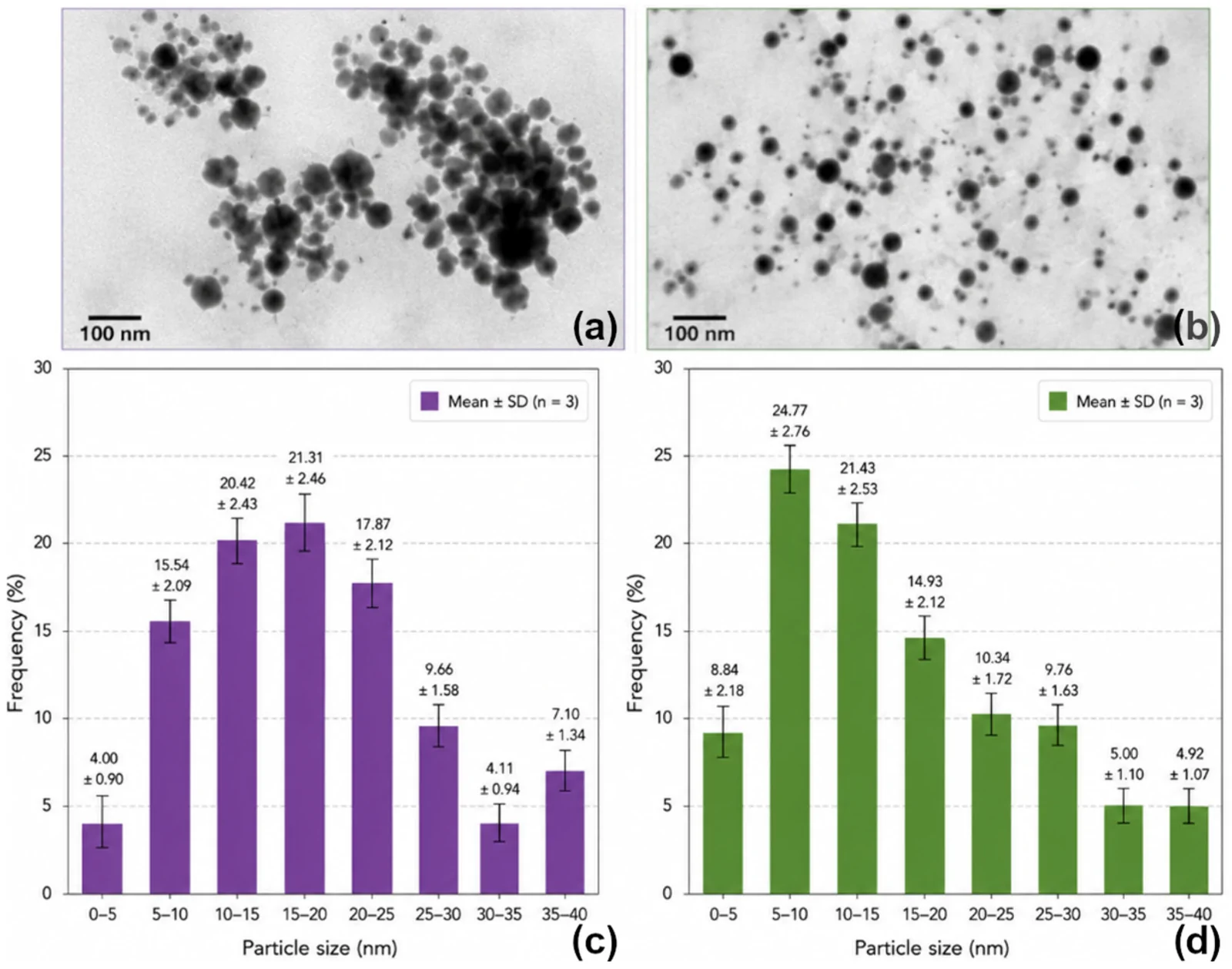
TEM images of AgNPs synthesized using *Lavandula angustifolia* extract (**a**) and particle size distribution (**c**), and TEM images of AgNPs synthesized using *Salvia rosmarinus* extract (**b**) and particle size distribution (**d**).

The morphology and size distribution of the synthesized AgNPs were analyzed using TEM micrographs and corresponding statistical evaluation. As shown in Figure 2, both samples exhibit predominantly spherical nanoparticles with a relatively narrow size distribution [30].

For the lavender-derived AgNPs, the particle size distribution is centered mainly within the range of 10–25 nm, with the highest frequency observed in the 15–20 nm interval (21%). The overall distribution indicates a relatively homogeneous population of nanoparticles with moderate polydispersity.

In contrast, the rosemary-derived AgNPs show a slightly broader size distribution, with the dominant fraction in the 5–15 nm range and a maximum frequency at 5–10 nm (25%). This suggests the formation of smaller nanoparticles compared to the lavender system, but with a wider distribution profile.

The particle size distribution was evaluated from TEM micrographs based on eight independent images for each sample. A total of 960 nanoparticles were analyzed for the lavender-derived AgNPs and 1199 nanoparticles for the rosemary-derived AgNPs.

#### 3.1.3. Surface Morphology (SEM)

**Figure 3:**
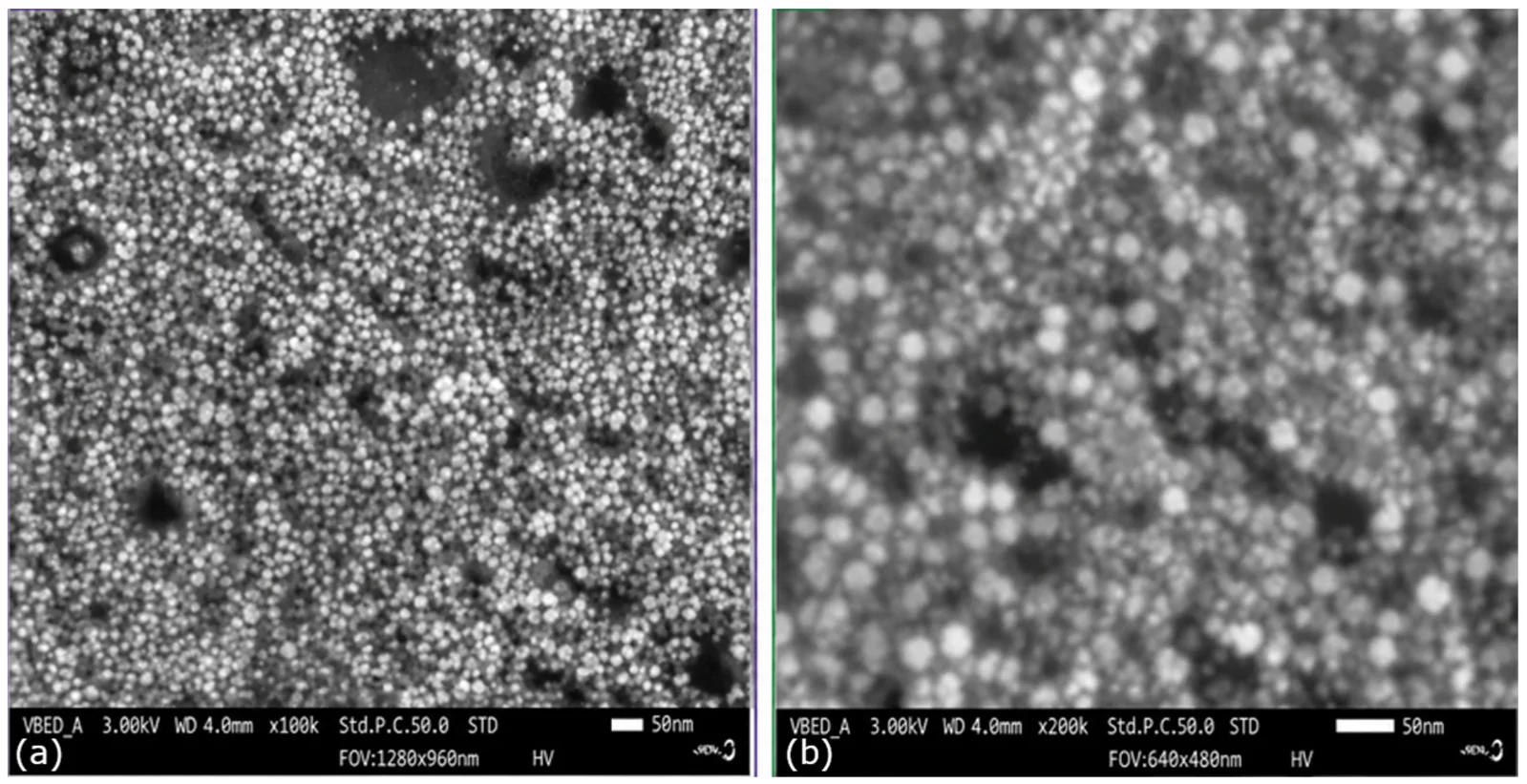
SEM images of AgNPs synthesized using (**a**) *Lavandula angustifolia* and (**b**) *Salvia rosmarinus* extracts.

The surface morphology of the synthesized AgNPs was further investigated using SEM, as shown in Figure 3. The obtained images reveal a high density of well-dispersed nanoparticles with predominantly spherical morphology.

For both samples, the nanoparticles appear relatively uniform in shape, forming densely packed structures without significant agglomeration. The lavender-derived AgNPs exhibit a slightly broader size distribution and occasional clustering, whereas the rosemary-derived nanoparticles appear more uniformly distributed across the surface.

The observed morphology is consistent with the results obtained from TEM and UV–Vis analyses, confirming the successful formation of nanoscale silver particles. The absence of large aggregates further supports the stabilizing effect of phytochemicals present in the plant extracts, which prevent excessive particle growth and aggregation.

#### 3.1.4. Elemental Composition (EDS)

**Figure 4:**
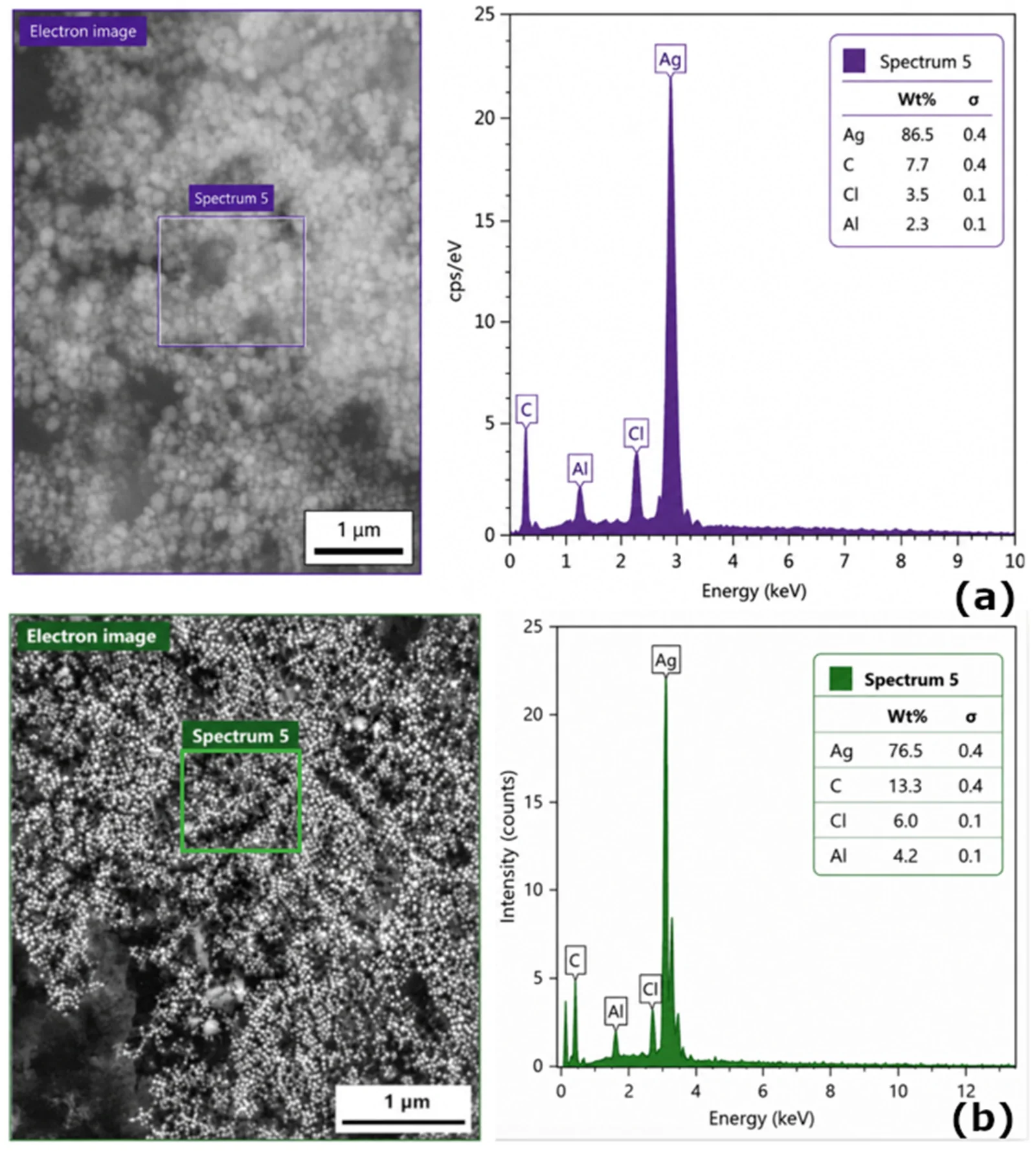
SEM images and corresponding EDS spectra of AgNPs synthesized using (**a**) *Lavandula angustifolia* and (**b**) *Salvia rosmarinus* extract.

The morphology and elemental composition of the synthesized AgNPs were further investigated using SEM coupled with EDS analysis (Figure 4). The SEM images confirm the formation of densely distributed nanoparticles with predominantly spherical morphology for both samples.

The corresponding EDS spectra reveal a dominant silver (Ag) peak at approximately 3 keV, confirming the successful formation of silver nanoparticles. In addition to silver, minor signals of carbon (C), chlorine (Cl), and aluminum (Al) were detected. The presence of carbon can be attributed to organic compounds originating from the plant extracts and the carbon-coated support used during analysis, while the aluminum and chlorine signals are likely associated with the sample holder and environmental background [29].

No additional peaks corresponding to impurities or undesired elements were observed, indicating the high purity of the synthesized AgNPs. The strong intensity of the Ag peak further confirms that silver is the main constituent of the analyzed nanoparticles.

Although oxygen-containing phytochemicals were expected to be present on the nanoparticle surface, no oxygen peak was observed in the EDS spectrum. This can be attributed to the limited sensitivity of EDS towards light elements and the very low amount of oxygen-containing organic molecules forming a thin capping layer on the AgNP surface. Their presence was, however, confirmed by FTIR through characteristic oxygen-containing functional groups.

#### 3.1.5. Hydrodynamic Size, Polydispersity Index, and ζ-Potential

Dynamic light scattering (DLS) measurements were performed in triplicate to further characterize the synthesized silver nanoparticles. Representative DLS intensity size distributions are presented in the Supplementary Materials (Figures S1 and S2). The DLS analysis revealed hydrodynamic diameters of 88.64 nm for AgNPs synthesized using *Lavandula angustifolia* extract (AgNPs-L) and 58.69 nm for AgNPs synthesized using *Salvia rosmarinus* extract (AgNPs-R). In both samples, a minor particle population with a hydrodynamic diameter of approximately 4.6 nm was also detected. The measured zeta potential values were −15.1 mV for AgNPs-L and −20.0 mV for AgNPs-R, while the corresponding polydispersity index (PDI) values were 0.308 and 0.426, respectively. Representative zeta potential distributions are presented in the Supplementary Materials (Figures S3 and S4). The larger hydrodynamic diameter observed for AgNPs-L compared with AgNPs-R may be attributed to differences in the phytochemical composition of the plant extracts, which can influence nanoparticle nucleation, growth, and the adsorption of phytochemicals onto the nanoparticle surface. Although both samples exhibited moderately negative zeta potential values, they remained stable under the storage conditions used in this study, demonstrating sufficient colloidal stability for the subsequent physicochemical characterization and biological experiments.

#### 3.1.6. FTIR Analysis

FTIR was performed to identify the functional groups present in the plant extracts and to evaluate their involvement in the biosynthesis of silver nanoparticles. Representative FTIR spectra of *Lavandula angustifolia* and *Salvia rosmarinus* extracts before and after AgNP synthesis are provided in the Supplementary Materials (Figure S5). For *L. angustifolia*, the O–H stretching band shifted from 3370 to 3400 cm^−1^, the C–O–H band from 1420 to 1390 cm^−1^, the C–O stretching band from 1260 to 1263 cm^−1^, and the glucose-related band from 1050 to 1052 cm^−1^, whereas the band at 2930 cm^−1^ remained unchanged. Similarly, for *S. rosmarinus*, the C=O stretching band shifted from 1720 to 1709 cm^−1^, the aromatic C=C vibration from 1603 to 1607 cm^−1^, and the N–H bending vibration from 1526 to 1530 cm^−1^, while the O–H (3354 cm^−1^), C–H (2927 cm^−1^), C–O (1264 cm^−1^) and C–O–C (1075 cm^−1^) bands remained essentially unchanged. In addition to these slight shifts, changes in the relative intensities of several absorption bands were observed after nanoparticle synthesis, indicating the participation of plant-derived phytochemicals in the reduction of Ag^+^ ions and the subsequent stabilization of the synthesized AgNPs. These observations are consistent with the proposed mechanism of green synthesis, in which polyphenols and other oxygen-containing biomolecules act as both reducing and capping agents.

#### 3.1.7. Proposed Mechanism of Green Synthesis

The formation of silver nanoparticles in the presence of plant extracts is governed by the redox activity of naturally occurring biomolecules. In this study, extracts obtained from *Lavandula angustifolia* and *Salvia rosmarinus* served as both reducing and stabilizing agents, enabling nanoparticle formation under mild conditions.

**Figure 5:**
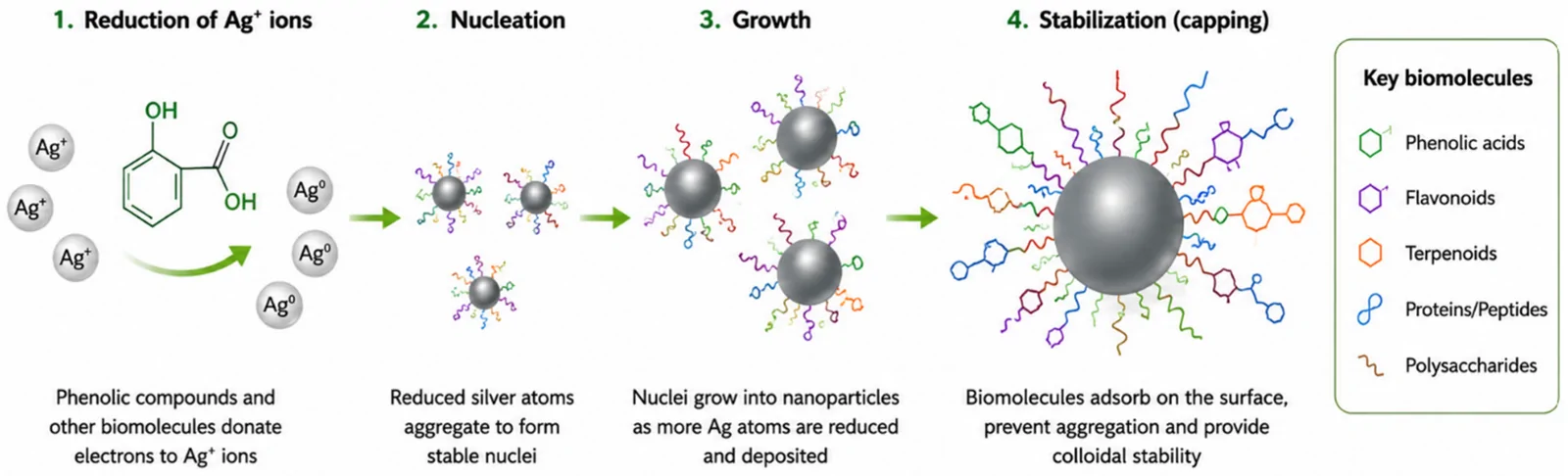
Mechanism of green synthesis of AgNPs using plant extracts.

The reduction of Ag^+^ ions is attributed primarily to phenolic compounds [22,31,32] and other redox-active constituents present in the extracts, which facilitate electron transfer and initiate nucleation processes (Figure 5). The subsequent growth of nanoparticles is influenced by the availability of these biomolecules, as well as by the reaction conditions such as temperature and extract concentration.

Simultaneously, organic compounds present in the extracts adsorb onto the surface of the forming nanoparticles, creating a protective layer that limits particle aggregation and contributes to colloidal stability. This dual role of plant-derived components allows for the controlled formation of AgNPs without the use of external chemical stabilizers [23,33].

Overall, the synthesis mechanism reflects a complex interplay between reduction kinetics and surface stabilization, which determines the final size, morphology, and stability of the nanoparticles.

Overall, the results demonstrate that both plant extracts act as effective reducing and stabilizing agents, enabling the formation of stable AgNPs with well-defined size and morphology. Differences in particle size and distribution between the two systems can be attributed to variations in the composition of bioactive compounds present in the respective plant extracts.

### 3.2. Antibacterial Activity of the Tested Samples

#### 3.2.1. Antibacterial Activity of Lavender- and Rosemary-Derived AgNPs

**Figure 6:**
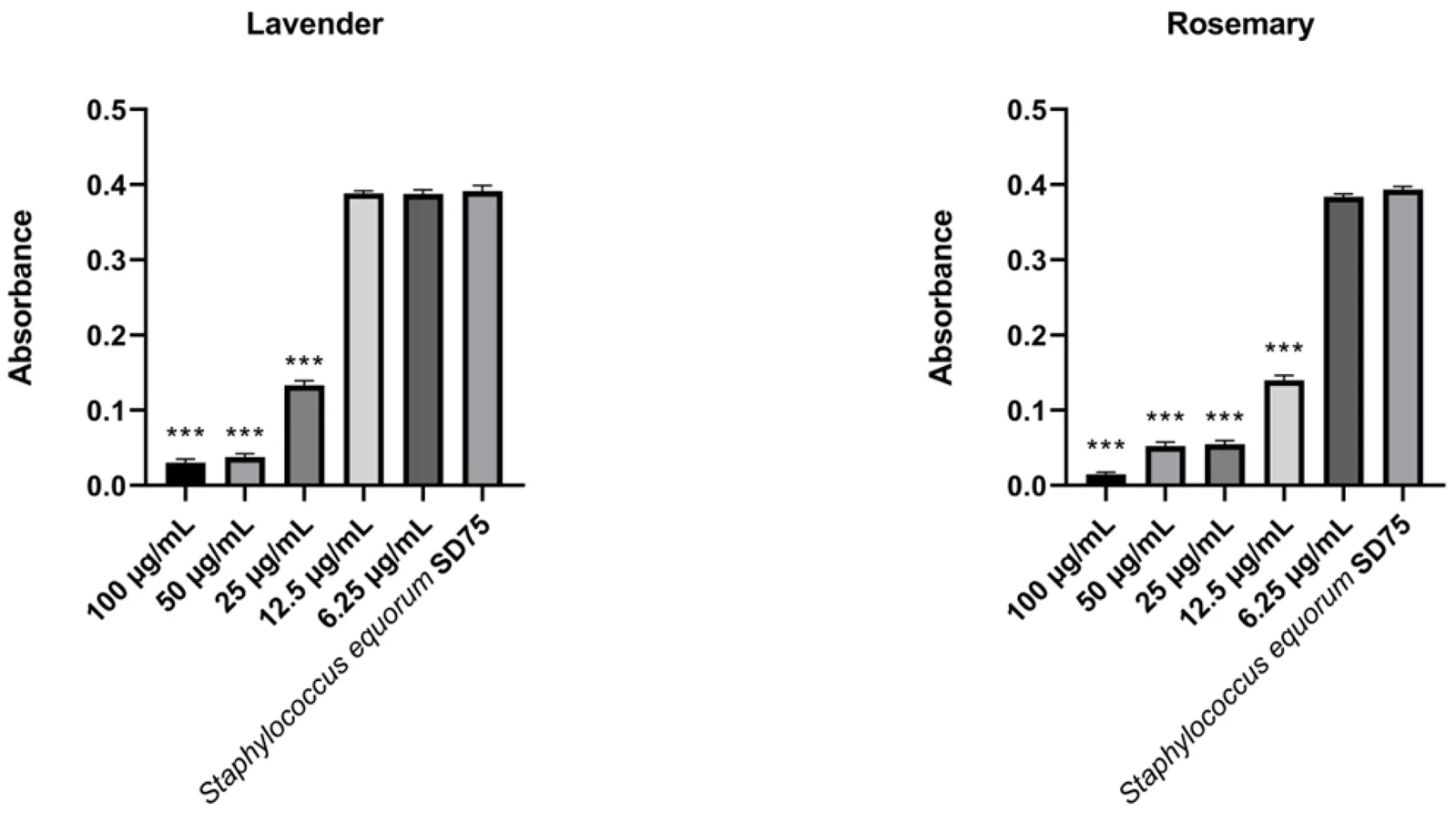
Antibacterial activity of AgNPs-L and AgNPs-R against *S. equorum.* Values are presented as mean ± SD (*n* = 3). *** *p* < 0.001.

The antibacterial activity of lavender- and rosemary-derived AgNPs (AgNPs-R, AgNPs-L) against the tested *S. equorum* strain differed between the two nanoparticle types. The MIC of AgNPs-L was determined to be 25 μg/mL, whereas AgNPs-R exhibited higher antibacterial activity with a lower MIC value of 12.5 μg/mL. Based on these results, subinhibitory concentrations used in subsequent experiments were selected as 12.5 μg/mL for AgNPs-L and 6.25 μg/mL for AgNPs-R (Figure 6). Statistical analysis also confirmed significant differences between the groups treated with AgNPs and the positive control. Detailed statistical results are presented in Supplementary Table S1.

#### 3.2.2. Antibacterial Activity of LEO and REO

**Figure 7:**
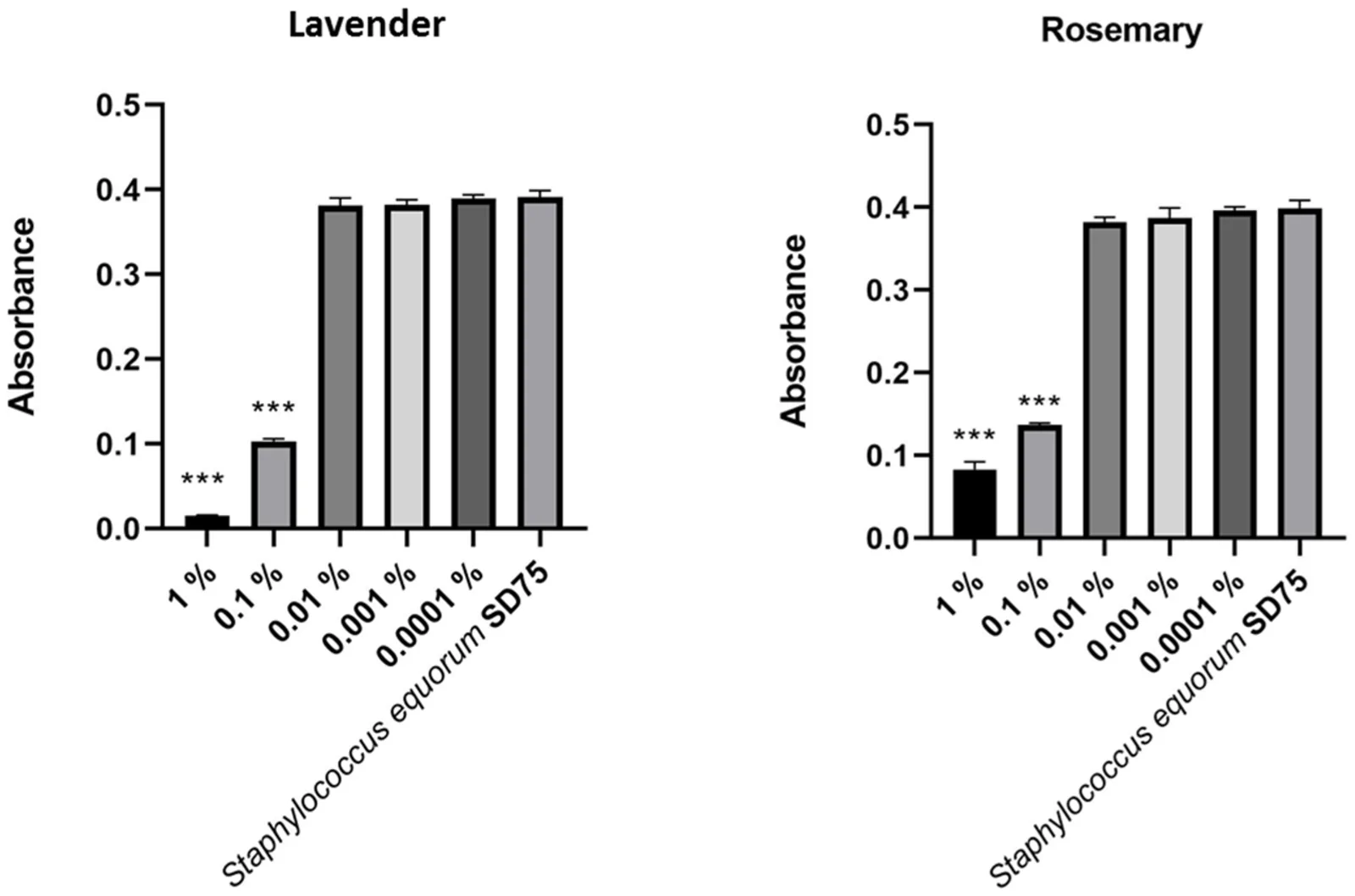
Antibacterial activity of LEO and REO against *S. equorum*. Values are presented as mean ± SD (*n* = 3). *** *p* < 0.001.

The MIC values of LEO and REO against the tested *S. equorum* strain were identical, reaching 0.1% (*v*/*v*). These values corresponded to approximately 0.0901% (*w*/*v*) for LEO (0.901 mg/mL) and 0.0929% (*w*/*v*) for REO (0.929 mg/mL). Based on the determined MIC values, subinhibitory concentrations of 0.01% (*v*/*v*) were selected for both essential oils in subsequent experiments (Figure 7). Statistical analysis is provided in Supplementary Table S2.

#### 3.2.3. Antibacterial Activity of Combinations of EOs, AgNPs, and AMP

**Figure 8:**
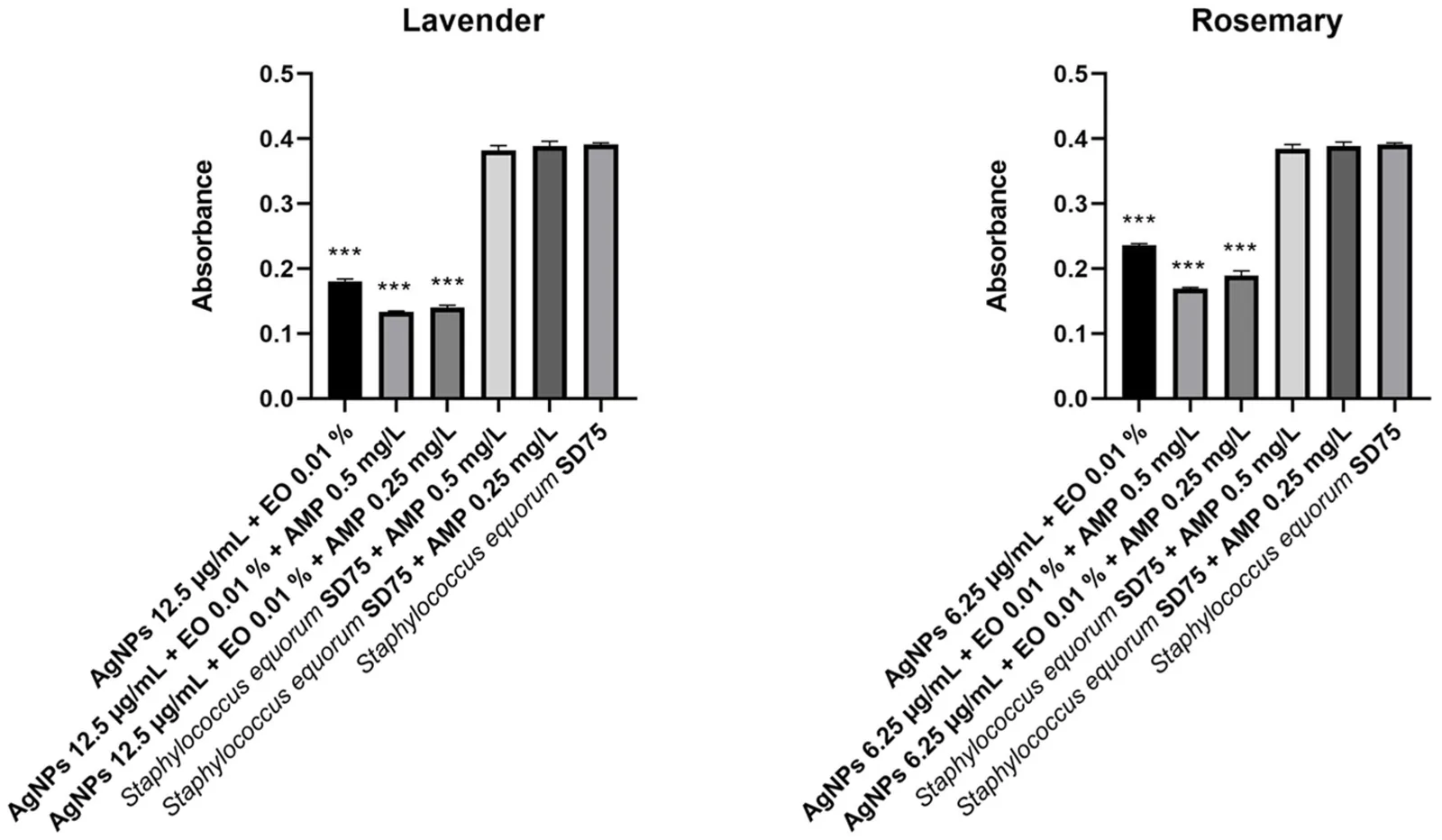
Antibacterial activity of combinations of EOs, AgNPs, and AMP against *S. equorum*. Values are presented as mean ± SD (*n* = 3). *** *p* < 0.001.

The antibacterial activity of the combinations was evaluated using several combination approaches. In all tested combinations, sub-MIC concentrations of AgNPs (12.5 μg/mL for AgNPs-L and 6.25 μg/mL for AgNPs-R), EOs (0.01%), and AMP (0.5 mg/L and 0.25 mg/L) were applied to assess their interaction effects on bacterial growth inhibition [21]. Both lavender- and rosemary-based combinations exhibited statistically significant antibacterial activity in all tested combinations (Figure 8). Statistical analysis is presented in Supplementary Table S3.

In lavender-based combinations, the application of AgNPs-L/LEO resulted in a pronounced reduction in absorbance, reaching 55.3% compared with AgNPs-L and 53.4% compared with LEO alone. The subsequent addition of AMP further decreased the absorbance values to 0.133 (0.5 mg/L) and 0.1346 (0.25 mg/L), corresponding to reductions of 63.9% and 63.5%, respectively, compared with AMP alone. The triple combination of AgNPs-L, LEO, and AMP demonstrated a stronger inhibitory potential than the AgNPs-L/LEO combination, with an increase in inhibition of 23.4% (0.5 mg/L) and 22.5% (0.25 mg/L).

For rosemary-based combinations, the AgNPs-R/REO combination decreased absorbance by 39.8% compared with AgNPs-R and 39.5% compared with REO alone. The addition of AMP resulted in a further reduction of absorbance to 0.158 (0.5 mg/L) and 0.185 (0.25 mg/L), representing decreases of 57.1% and 49.8%, respectively, compared with AMP alone. The triple combination of AgNPs-R, REO, and AMP exhibited a stronger inhibitory effect than the AgNPs-R/REO combination, with increases in inhibition of 31.6% (0.5 mg/L) and 20.0% (0.25 mg/L).

All tested combinations were prepared using sub-MIC concentrations of AgNPs, EOs, and AMP. Despite the use of subinhibitory concentrations, the combinations resulted in reduced bacterial growth compared with the individual components. The interaction between the compounds, evaluated by FICI analysis, showed that the AgNPs/EO combination reached an FICI value of 0.501, indicating a borderline additive effect. For the AgNPs/EO/AMP triple combination, FICI values of 0.751 at 0.25 mg/L AMP and 1.001 at 0.5 mg/L AMP were observed, corresponding to an additive to indifferent interaction profile depending on the AMP concentration. Nevertheless, the observed reduction in bacterial growth at sub-MIC concentrations suggests that the combined application of these compounds may enhance antibacterial activity compared with their individual effects.

### 3.3. Antibiofilm Activity of the Tested Samples

#### 3.3.1. Antibiofilm Activity of AgNPs-L and AgNPs-R

**Figure 9:**
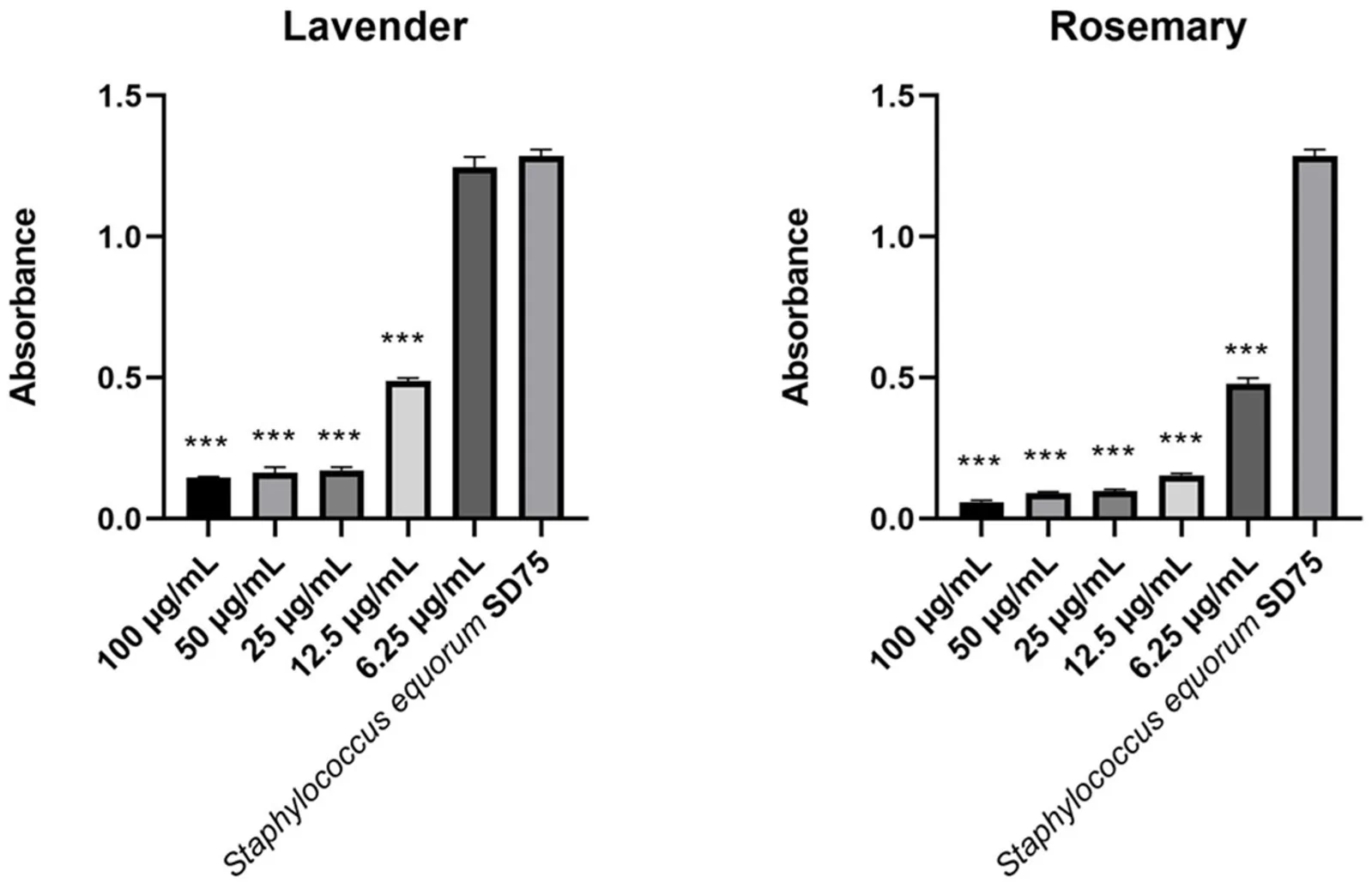
Antibiofilm activity of AgNPs-L and AgNPs-R against *S. equorum*. Values are presented as mean ± SD (*n* = 3). *** *p* < 0.001.

Antibiofilm activity of AgNPs was evaluated at concentrations corresponding to those used for the assessment of antibacterial activity. Inhibition of biofilm formation by *S. equorum* was observed at concentrations of 12.5 μg/mL for AgNPs-L and 6.25 μg/mL for AgNPs-R. Compared with their antibacterial activity, the antibiofilm effect was observed at concentrations one two-fold dilution step lower, indicating a higher susceptibility of biofilm formation processes to the action of the tested nanoparticles (Figure 9). Statistical analysis is provided in Supplementary Table S4.

#### 3.3.2. Antibiofilm Activity of LEO and REO

**Figure 10:**
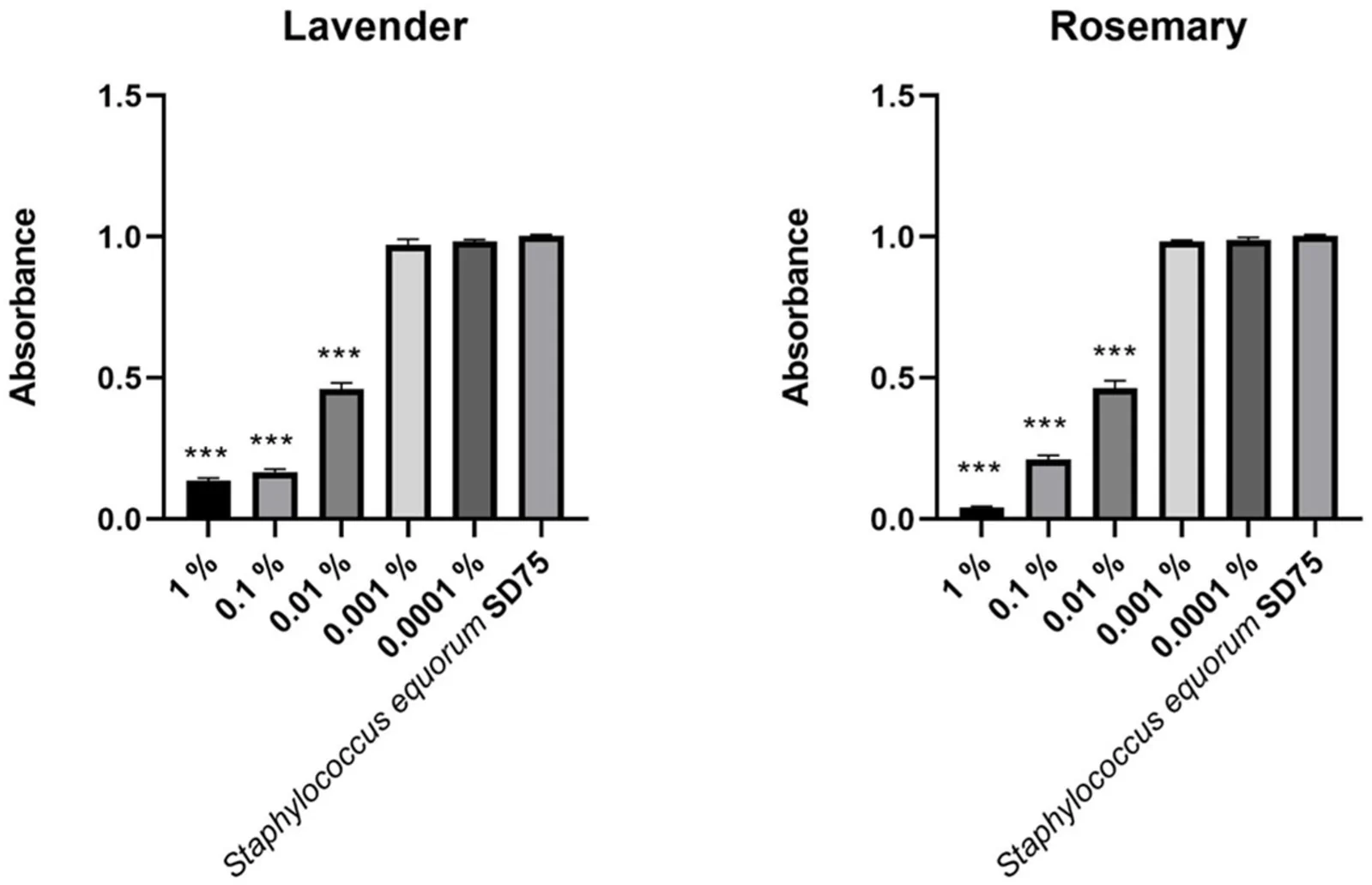
Antibiofilm activity of EOs against *S. equorum*. Values are presented as mean ± SD (*n* = 3). *** *p* < 0.001.

The antibiofilm activity of LEO and REO against the tested *S. equorum* strain was comparable. A highly significant inhibition of biofilm formation was observed at a concentration of 0.01% (*v*/*v*), corresponding to approximately 0.00901% (*w*/*v*) for LEO and 0.00929% (*w*/*v*) for REO. A statistically significant effect was maintained even at 0.001% (*v*/*v*), corresponding to approximately 0.000901% (*w*/*v*) for LEO and 0.000929% (*w*/*v*) for REO. Compared with the MIC values, the minimum biofilm inhibitory concentration (MBIC) was one to two logarithmic dilutions lower, indicating a high susceptibility of biofilm formation to the action of the tested essential oils (Figure 10). Statistical analysis is presented in Supplementary Table S5.

#### 3.3.3. Antibiofilm Activity of Combinations of EOs, AgNPs, and AMP

**Figure 11:**
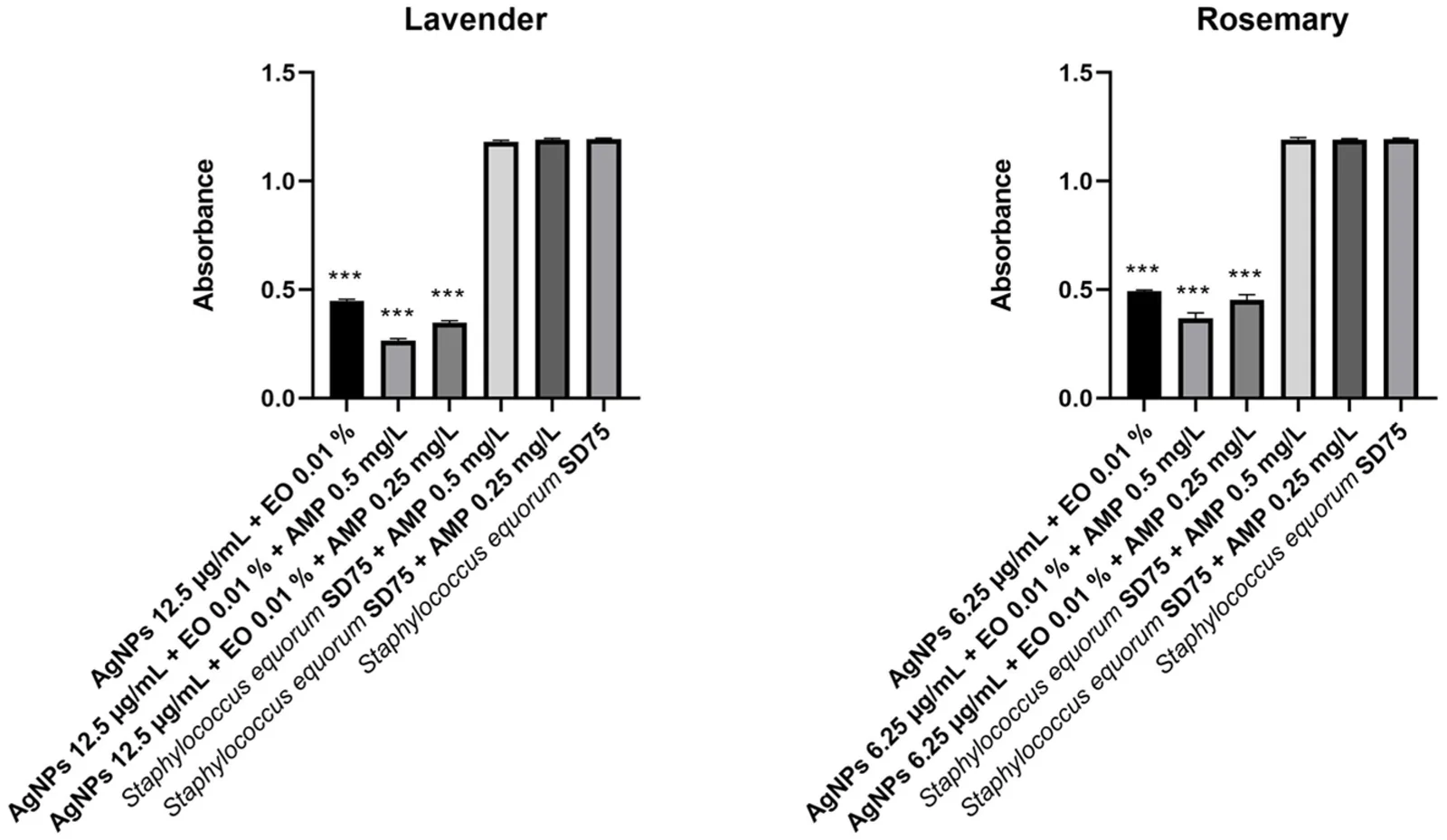
Antibiofilm activity of combinations of EOs, AgNPs, and AMP against *S. equorum*. Values are presented as mean ± SD (*n* = 3). *** *p* < 0.001.

The antibiofilm activity of the combinations was evaluated using the same experimental design as for antibacterial activity, applying sub-MIC to assess their interaction effects on biofilm formation inhibition. For both lavender- and rosemary-based combinations, all three tested variants demonstrated a highly significant antibiofilm effect (Figure 11). Statistical analysis is provided in Supplementary Table S6.

In lavender-based samples, the combination of AgNPs-L/LEO resulted in only a negligible reduction in absorbance, corresponding to a 4.2% decrease compared with AgNPs-L and 0.2% compared with LEO alone. However, the subsequent addition of AMP caused a marked reduction in absorbance to 0.254429 (0.5 mg/L) and 0.332571 (0.25 mg/L), representing decreases of 78.1% and 72.2%, respectively, compared with AMP alone. The triple combination of AgNPs-L, LEO, and AMP exhibited a 43.1% (0.5 mg/L) and 25.6% (0.25 mg/L) higher inhibitory effect compared with the AgNPs-L/LEO combination.

Similarly, in rosemary-based samples, the AgNPs-R/REO combination did not result in a statistically significant reduction in absorbance, with decreases of only 5.1% compared with AgNPs-R and 1.7% compared with REO alone. Following AMP addition, a pronounced reduction in absorbance was observed, reaching 0.355714 (0.5 mg/L) and 0.418143 (0.25 mg/L), corresponding to decreases of 69.4% and 65.0%, respectively. The triple combination of AgNPs-R, REO, and AMP demonstrated a 24.0% (0.5 mg/L) and 10.7% (0.25 mg/L) higher inhibitory effect compared with the AgNPs-R/REO combination.

Overall, the obtained results demonstrate the pronounced antibiofilm activity of the triple combination of AgNPs-R, EOs, and AMP, which exhibited the highest inhibitory effects compared with the dual combinations and individual components.

### 3.4. Biofilm-Eradicating Activity of Tested Samples

#### 3.4.1. Biofilm-Eradicating Activity of AgNPs-L, AgNPs-R, LEO and REO

**Figure 12:**
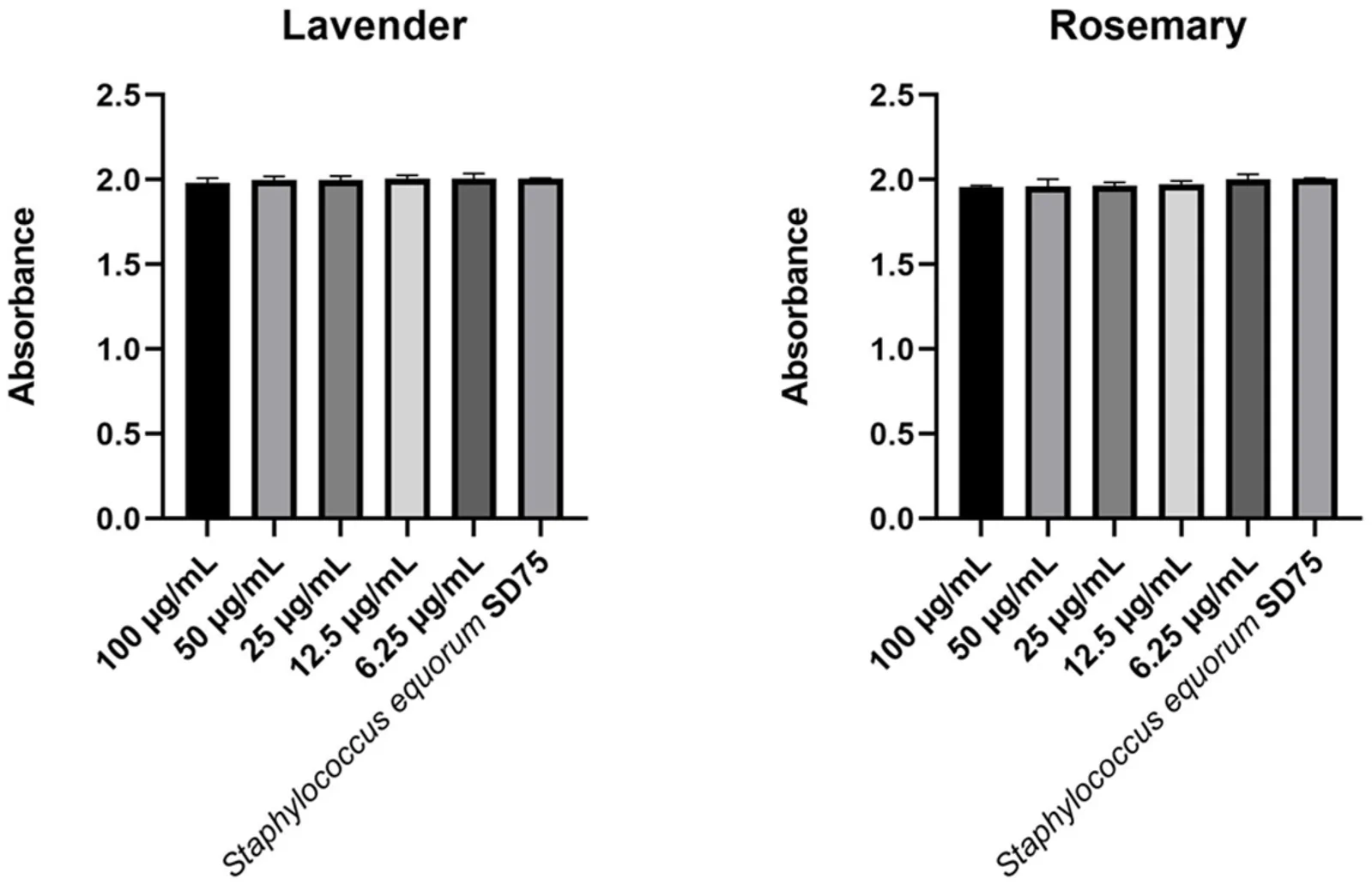
Effect of AgNPs on biofilm eradication against *S. equorum*. Values are presented as mean ± SD (*n* = 3).

**Figure 13:**
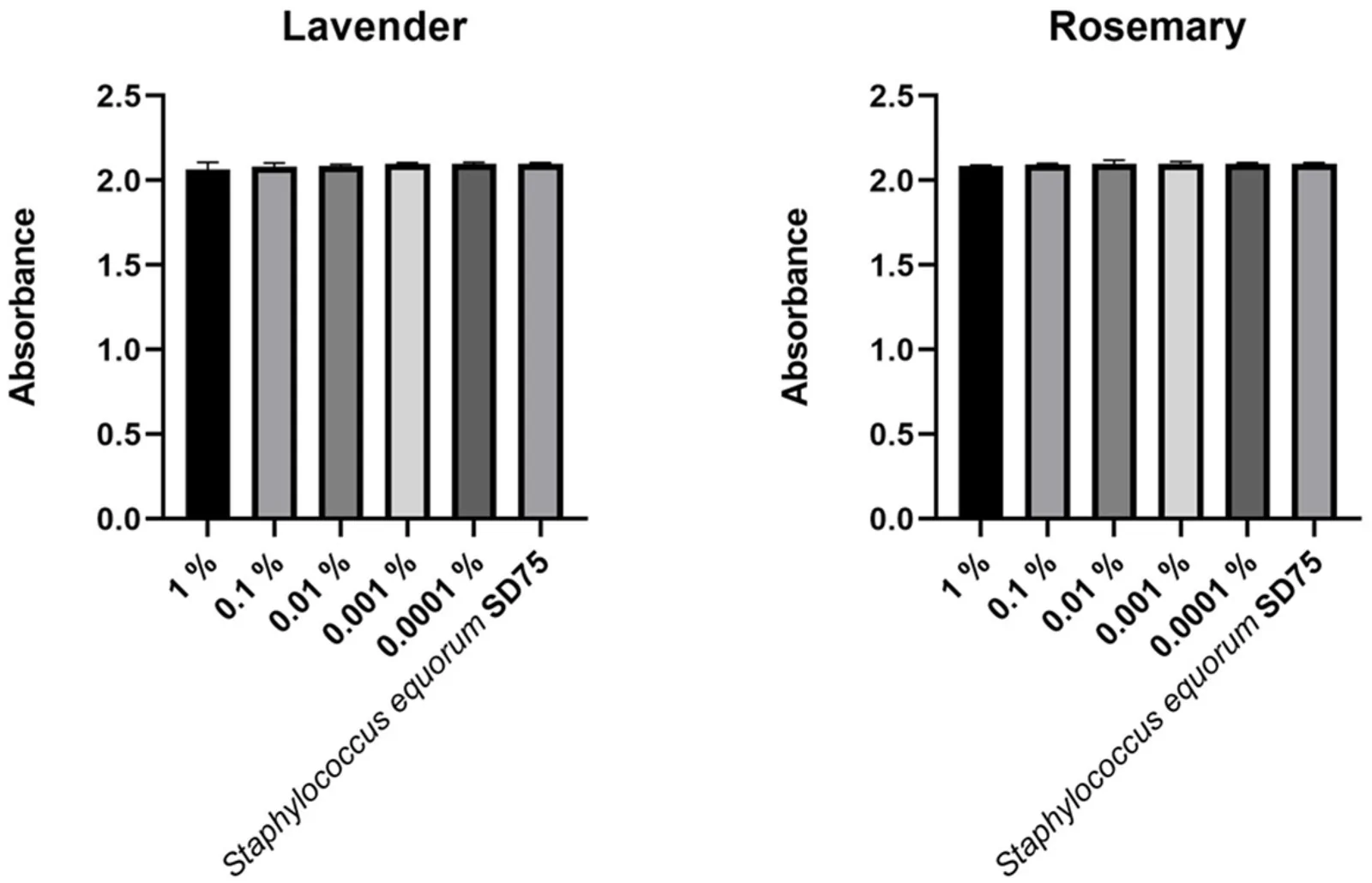
Effect of EOs on biofilm eradication against *S. equorum*. Values are presented as mean ± SD (*n* = 3).

The final step of the experiments was to evaluate the ability of the tested samples to disrupt the preformed biofilm of the biofilm-producing *S. equorum* strain. None of the tested AgNPs and EO concentrations demonstrated a significant reduction in the amount of established biofilm, and no minimum biofilm eradication concentration (MBEC) was determined (Figure 12 and Figure 13). Statistical analysis is provided in Supplementary Tables S7 and S8.

#### 3.4.2. Biofilm-Eradicating Activity of Combinations of EOs, AgNPs, and AMP

**Figure 14:**
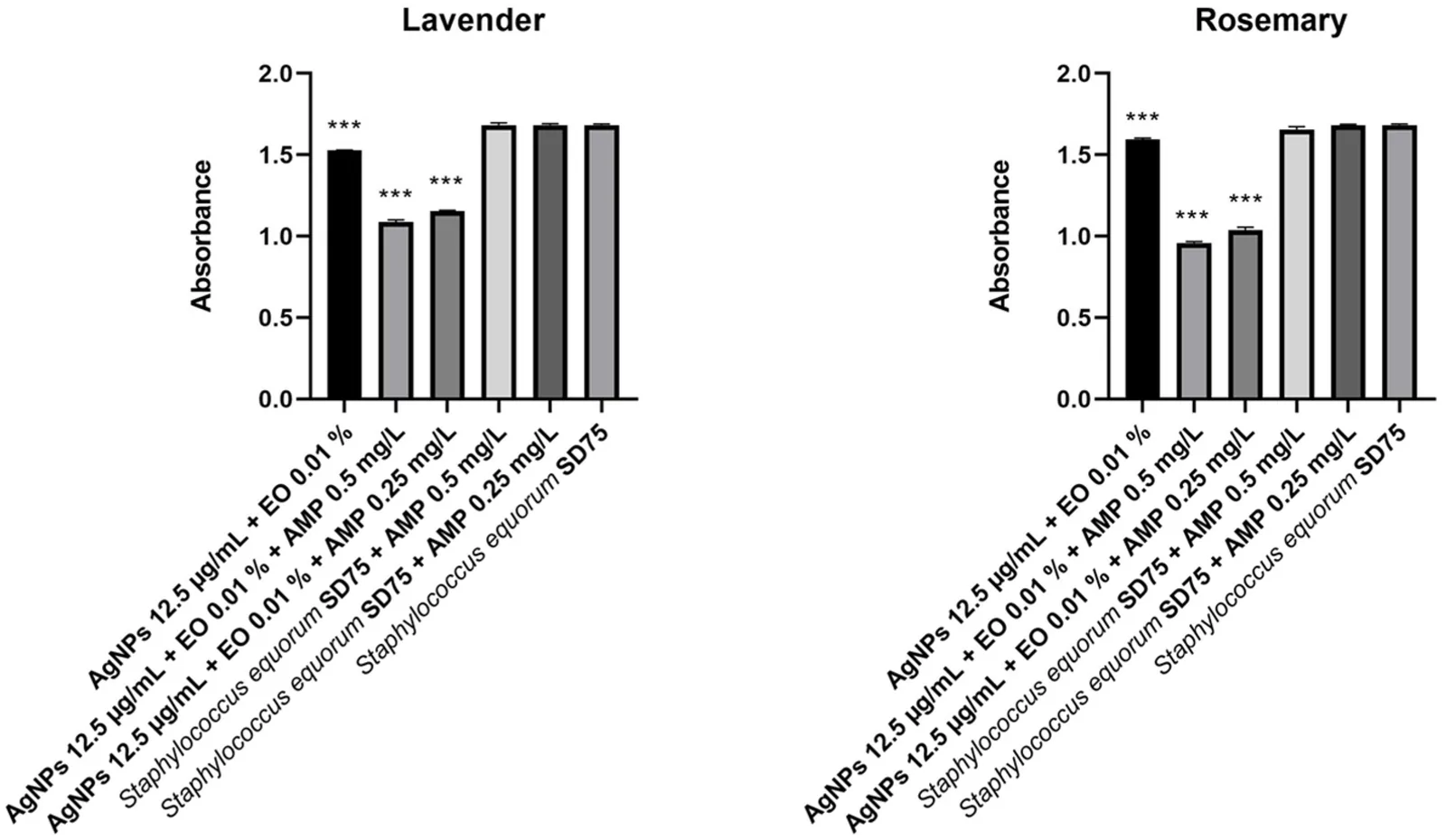
Effect of combinations of EOs, AgNPs, and AMP on biofilm eradication against *S. equorum*. Values are presented as mean ± SD (*n* = 3). *** *p* < 0.001.

In contrast to the individual compounds, the combined treatments demonstrated a significant eradication effect against the preformed biofilm. For both lavender- and rosemary-based combinations, all three tested variants exhibited a statistically significant biofilm eradication activity (Figure 14). Detailed statistical analysis is provided in Supplementary Table S9.

In lavender-based samples, the combination of AgNPs-L/LEO resulted in a reduction in absorbance of 20.9% compared with AgNPs-L and 25.0% compared with LEO alone. The addition of AMP to the AgNPs-L/LEO combination further decreased absorbance to 1.05188 (0.5 mg/L) and 1.13748 (0.25 mg/L), corresponding to reductions of 37.3% and 32.9%, respectively, compared with AMP alone. The triple combination of AgNPs-L, LEO, and AMP exhibited a 33.1% (0.5 mg/L) and 27.7% (0.25 mg/L) higher eradication effect compared with the AgNPs-L/LEO combination.

Similarly, in rosemary-based samples, the AgNPs-R/REO combination reduced absorbance by 19.5% compared with AgNPs-R and 23.3% compared with REO alone. Following AMP addition, a further reduction in absorbance was observed, reaching 0.961 (0.5 mg/L) and 1.06098 (0.25 mg/L), representing decreases of 42.7% and 39.1%, respectively, compared with AMP alone. The triple combination of AgNPs-R, REO, and AMP demonstrated a 39.9% (0.5 mg/L) and 33.7% (0.25 mg/L) higher eradication effect compared with the AgNPs-R/REO combination.

Overall, these findings confirm the effectiveness of the tested combinations in promoting eradication of the preformed biofilm, whereas the individual components alone did not demonstrate any eradication activity.

### 3.5. Summary of the Main Findings

Based on the results obtained, the biological activities (antibacterial, antibiofilm, and biofilm eradication activities) of individual compounds and their combinations were evaluated against the clinically relevant, resistant, and biofilm-forming *S. equorum* strain. For a comprehensive comparison of their efficacy based on effective concentrations, the summarized results are presented in Table 4 and Table 5.

## 4. Discussion

The increasing prevalence of AMR, together with the ability of bacteria to establish persistent biofilms, represents a major challenge to effective antimicrobial therapy and highlights the need for novel strategies to enhance the efficacy of existing antimicrobial agents. In this context, NASM have emerged as relevant models for evaluating alternative antimicrobial approaches due to their resistance potential and pronounced biofilm-forming capacity. Biosynthesized AgNPs and plant-derived EOs have attracted considerable attention as promising antimicrobial agents because of their multiple mechanisms of action and their potential to potentiate the activity of conventional antibiotics. Accordingly, the present study evaluated the antibacterial, antibiofilm, and biofilm-eradicating activities of AgNPs-L and AgNPs-R, their corresponding EOs, and their combinations with AMP against a multidrug-resistant, biofilm-forming *S. equorum* strain.

Numerous studies have demonstrated the pronounced antistaphylococcal activity of plant-mediated biosynthesized AgNPs. Nevertheless, reported MIC values vary substantially depending on the plant source, synthesis method, nanoparticle physicochemical properties, particularly size and morphology, and the bacterial strain tested [7,34,35,36,37]. In our study, the minimum inhibitory concentration of antibacterial activity was determined to be 12.5 µg/mL for AgNPs-R and 25 µg/mL for AgNPs-L, which is consistent with values published for similarly prepared biogenic nanoparticles.

For AgNPs-L, Onem et al. reported an MIC value of up to 250 µg/mL, which is approximately ten-fold higher than the value obtained in the present study [38]. This discrepancy may be associated with differences in nanoparticle synthesis procedures, the phytochemical composition of the plant extract used, nanoparticle size and morphology, as well as variations in the susceptibility of the tested bacterial strains. In contrast, a study employing green-synthesized AgNPs prepared using *Lavandula angustifolia* essential oil reported an MIC of only 8 µg/mL [39], highlighting the potential influence of surface functionalization by bioactive compounds derived from essential oils. Furthermore, Ödemiş et al. demonstrated complete inhibition of staphylococcal growth using AgNPs-L [40]. Similarly, AgNPs synthesized from *Salvia rosmarinus* have repeatedly exhibited strong antistaphylococcal activity, with reported MIC values ranging from 12 to 50 µg/mL [41,42]. Ahmad et al. further demonstrated that the conversion of rosemary phytochemicals into biogenic AgNPs significantly enhanced their antimicrobial activity compared with the corresponding plant extract alone [13]. The slightly higher antibacterial activity of AgNPs-R observed in our study may be attributed to differences in the phytochemical composition of the plant material, particularly the abundance of phenolic compounds, including rosmarinic acid, which may contribute to nanoparticle reduction, stabilization, and biological activity [43,44]. Comparable low MIC values have also been reported for biogenic AgNPs synthesized from other plant species, including *Convolvulus arvensis* (12.5–25 µg/mL), *Azanza garckeana* (12.5–25 µg/mL), and *Eichhornia crassipes* (15 µg/mL), suggesting that plant-mediated synthesis represents a reproducible approach for the generation of highly effective antimicrobial nanoparticles [45,46,47]. However, it should be noted that several studies have evaluated antibacterial activity exclusively using the disk diffusion method and reported inhibition zone diameters, which limits direct comparison with studies determining MIC values by broth microdilution assays [48,49].

In the present study, the antibiofilm activity of biosynthesized AgNPs was observed at concentrations one two-fold dilution step lower than their corresponding MIC values, specifically at 12.5 µg/mL for AgNPs-L and 6.25 µg/mL for AgNPs-R. This observation is consistent with several previously published studies. Nada et al. reported biofilm inhibition at a concentration corresponding to ½ MIC, indicating that suppression of biofilm formation may occur at concentrations below those required for complete inhibition of planktonic growth [50]. Similarly, Jaiswal and Mishra demonstrated a significant antibiofilm effect of AgNPs at concentrations as low as 10 µg/mL [51], while Hamid et al. also reported lower MBIC values compared with MIC values [52]. The lower MBIC observed compared with MIC may be explained by the ability of sub-MIC concentrations of AgNPs to interfere with early stages of biofilm development, particularly bacterial adhesion to surfaces and regulatory pathways involved in biofilm formation, including quorum sensing, before complete inhibition of planktonic growth is achieved [50]. A significant inhibition of biofilm formation was also reported by Gopinath et al., although their evaluation was based on morphological assessment of biofilm thickness using microscopic techniques [53]. Similarly, Hosnedlova et al. demonstrated a pronounced antibiofilm effect of AgNPs at relatively low concentrations, with an MBIC value of 20 µg/mL [54].

In contrast, no eradication activity against preformed biofilm was observed in our study, even at the highest tested concentration of 100 µg/mL. This finding is consistent with previous reports indicating that eradication of established biofilms is considerably more challenging than prevention of biofilm formation and generally requires concentrations several-fold higher than the MIC. Wahab et al. achieved approximately 65% biofilm reduction at 65 µg/mL, which represented a concentration several times higher than the MIC of the tested AgNPs [55]. Similarly, Madhusudanan et al. reported biofilm eradication concentrations ranging from 40 to 175 µg/mL depending on the type of biogenic AgNPs and the bacterial isolate evaluated [56]. These findings highlight that the mechanisms underlying inhibition of initial biofilm formation differ substantially from those required for disruption of mature biofilm structures, where the extracellular polymeric substance (EPS) matrix represents an effective barrier limiting the penetration and activity of antimicrobial agents.

Essential oils represent complex mixtures of plant-derived bioactive compounds with well-documented antimicrobial properties. Their activity is associated with multiple mechanisms, including disruption of bacterial membrane integrity, interference with cellular metabolic processes, and inhibition of both planktonic and biofilm-associated bacterial populations [57,58]. Due to these properties, EOs are considered promising natural antimicrobial agents with potential applications as alternatives or complementary approaches to conventional antibiotic therapy [59]. Therefore, one of the objectives of the present study was to evaluate the antibacterial, antibiofilm, and biofilm-eradicating activities of rosemary and lavender essential oils against a biofilm-forming *S. equorum* strain.

Based on our previous experience and published data regarding the antimicrobial activity of various EOs, a concentration range based on ten-fold serial dilutions was selected for the evaluation of REO [16]. The bacteriostatic activity of REO against *Staphylococcus* spp. has been reported in several studies, with MIC values ranging from 0.025 to 6.25 mg/mL. The MIC determined in our study (0.929 mg/mL) falls within this previously reported range [60,61,62,63]. De Moraes et al. reported an MIC of 0.13% against MDR *S. aureus* strains, which is comparable to the MIC obtained in our study for *S. equorum* (0.1% (*v*/*v*)). However, the antimicrobial activity of REO is not limited to staphylococci. Several studies have demonstrated its efficacy against other multidrug-resistant pathogens, including representatives of the ESKAPE group (*Enterococcus faecium*, *Staphylococcus aureus*, *Klebsiella pneumoniae*, *Acinetobacter baumannii*, *Pseudomonas aeruginosa*, and *Enterobacter* spp.). Cintra et al. reported MIC values ranging from 1.88 to 3.75 mg/mL against *A. baumannii*, *Ps. aeruginosa*, and *K. pneumoniae* [64]. Nevertheless, most available studies have focused primarily on *S. aureus*, while NASM remain comparatively underexplored in this context.

The results obtained from the evaluation of REO antibiofilm and biofilm-eradicating activities are consistent with previously published findings. In our study, the MBIC was determined at a concentration one logarithmic dilution lower than the MIC. Similar observations were reported by Aniba et al., who demonstrated a significant antibiofilm effect of EOs against *S. aureus* at subinhibitory concentrations, with this effect exceeding that of antibiotics in some isolates [65]. Abdallah et al. investigated the activity of several EOs, including REO, against multidrug-resistant *S. aureus* (MRSA) and demonstrated pronounced antibacterial and antibiofilm effects; however, the ability to eradicate preformed biofilm was considerably limited. Comparable results have been reported in other studies, where biofilm-eradicating activity was observed only in a limited number of isolates, and no significant association was found between MIC values and the ability to eradicate established biofilms [66]. These findings are further supported by Bowbe et al., who demonstrated differences between MBIC and MBEC values. Their results showed that eradication of preformed biofilm required higher concentrations than inhibition of biofilm formation [67]. A similar pattern was observed in our study, where REO effectively inhibited planktonic growth and biofilm formation but failed to eradicate mature biofilm structures. These findings suggest that the mechanisms responsible for inhibition of bacterial growth and prevention of biofilm development may differ from those required for disruption and removal of established biofilms.

The antibacterial activity of LEO was lower compared with REO, which is consistent with findings reported by Stamova et al., who determined an MIC of 5% (*v*/*v*) against *S. aureus* [68]. However, most other studies have reported MIC values ranging from 0.25 to 2% (*v*/*v*), depending on the bacterial isolate tested [69,70,71]. In the present study, the MIC of LEO was determined as 0.1% (*v*/*v*), which may be partially attributed to the use of ten-fold serial dilutions. Similar to REO, most available studies investigating LEO have focused primarily on *S. aureus*, including multidrug-resistant MRSA strains.

The MBIC of LEO was also determined at a concentration one logarithmic dilution lower than its MIC, in agreement with findings reported by Aniba et al. [65]. In contrast, Brozyna et al. did not observe eradication of staphylococcal biofilms even at the highest tested LEO concentration (50% (*v*/*v*)) [70]. Truong et al. reported that achieving 90% biofilm eradication required increasing MIC values by four- to eight-fold [72]. Therefore, the lack of significant biofilm-eradicating activity observed for LEO in our study is consistent with previous reports.

To ensure homogeneous dispersion of hydrophobic components, a 10% (*v*/*v*) stock solution of essential oils in polysorbate 20 was prepared according to validated methodological procedures, and subsequently used to prepare working concentrations in mBHI medium [73]. This approach enabled the formation of a stable emulsion while minimizing the final surfactant concentration in the test wells. Spadini et al. demonstrated that polysorbate 20 is biologically inert, stabilizes volatile components of lavender and rosemary oils, and does not interfere with their antimicrobial activity against staphylococci, thereby improving the reproducibility of experimental results [74]. Furthermore, Dávila-Rodríguez et al. reported a 20–75% higher antibacterial activity of emulsified EOs compared with non-emulsified formulations, which was attributed to improved dispersion and stability during testing [75].

The variability in bacterial susceptibility to EOs may be influenced by several factors, including differences in the chemical composition of oils associated with the geographical origin of the plant material [76], as well as strain-specific variations among staphylococcal isolates. Compared with AgNPs, EOs required higher concentrations to achieve comparable biological effects.

The aim of this study was to evaluate the interaction potential of AgNPs and EOs combined with AMP at subinhibitory concentrations against a biofilm-forming *S. equorum* strain. Considering the increasing occurrence of beta-lactam-resistant *S. equorum*, AMP was selected as a model antibiotic to assess the potential restoration of bacterial susceptibility within combined treatment systems [77]. The tested AgNPs/EO/AMP triple combinations demonstrated pronounced inhibitory effects on bacterial growth, reflected by a significant reduction in optical density compared with untreated controls. However, FICI analysis indicated predominantly additive interactions at the applied subinhibitory concentrations, specifically 12.5 µg/mL/0.901 mg/mL/0.25–0.5 mg/L for the lavender-based combination and 6.25 µg/mL/0.929 mg/mL/0.25–0.5 mg/L for the rosemary-based combination. Compared with AMP alone (0.25 and 0.5 mg/L), the combinations resulted in enhanced inhibition of bacterial growth, reaching 63.9% and 63.5% for the lavender-based system and 57.1% and 49.8% for the rosemary-based system. Regarding biofilm-associated activity, the combinations significantly inhibited biofilm formation, with inhibition rates of 78.1% and 72.2% for lavender and 69.4% and 65.0% for rosemary. Moreover, eradication of preformed biofilm reached 33.1% and 27.7% for the lavender-based combination and 39.9% and 33.7% for the rosemary-based combination.

The synergistic activity between nanoparticles and β-lactam antibiotics is frequently attributed to disruption of bacterial membranes and induction of reactive oxygen species (ROS) generation, resulting in increased cellular permeability and enhanced intracellular availability of antibiotics [78,79]. Additional mechanisms may involve interference with bacterial enzymes responsible for antibiotic inactivation. Ayubee et al. reported a one-fold reduction in AMP MIC when combined with AgNPs, which is consistent with our findings, as an enhancing effect was observed even at subinhibitory concentrations. Similar trends have also been described for MBIC and MBEC parameters [33,80]. Synergistic interactions between nanoparticles, essential oils, and antibiotics have been demonstrated in several studies, where combined treatments resulted in reduced MIC values and suppression of biofilm matrix formation [81]. Hwang et al. and Cui et al. confirmed enhanced antibiotic activity when combined with EOs against MRSA [71], which was also reported by Stamova et al. [68]. Furthermore, Meroni et al. demonstrated synergistic effects between AgNPs and EOs against multidrug-resistant *S. pseudintermedius* strains [82]. Although the efficacy of these combinations may vary depending on the chemical composition of EOs and the tested microorganism [83], their ability to modulate antimicrobial activity through multiple complementary mechanisms is increasingly recognized. The lack of standardized FICI-based evaluation in many studies limits direct quantitative comparison of synergistic or additive interactions, further emphasizing the importance of studies employing this approach.

The available literature has predominantly focused on dual combinations, whereas the effects of triple antimicrobial systems remain insufficiently explored. In the present study, the highest efficacy was observed for the AgNPs/EO/AMP triple combination, particularly regarding antibacterial, antibiofilm, and, most notably, biofilm-eradicating activity, which was achieved despite the use of subinhibitory concentrations of all components. In contrast, individual compounds and their dual combinations were unable to eradicate preformed *S. equorum* biofilm. This effect may be associated with the proposed disruption of the extracellular polymeric substance (EPS) matrix by EOs, which could facilitate deeper penetration of AgNPs and AMP into biofilm layers. Subsequent combined action may increase bacterial susceptibility; however, the precise mechanisms underlying these interactions require further experimental validation [84].

From an applicational perspective, the use of an NASM strain of *S. equorum* represents an important aspect of this study, as this species may act as an opportunistic pathogen with the potential for persistent biofilm-associated colonization [85]. The obtained results further suggest that antimicrobial combinations may enable the reduction in effective antibiotic concentrations while maintaining or enhancing antibacterial activity through the contribution of multiple bioactive components [86]. Overall, these findings support the potential of AgNPs/EOs/AMP combinations as a promising strategy for controlling multidrug-resistant and biofilm-forming bacteria through a multifaceted mechanism of action. In agreement with previous reports, such nano-phytosystems may exhibit a dual-stage antibiofilm effect, where early stages involve inhibition of bacterial adhesion and biofilm biomass formation, whereas established biofilms may be disrupted through EPS destabilization and subsequent impairment of metabolically active persistent cells [87,88].

Although FICI analysis did not demonstrate a clear synergistic interaction, the obtained results remain biologically relevant, as the application of sub-MIC concentrations resulted in enhanced antimicrobial efficacy and suggested potential resensitization of bacteria to ampicillin. However, the practical application of essential oils may be limited by their volatility, susceptibility to environmental factors, and instability of some bioactive constituents [89]. To overcome these limitations, advanced formulation strategies, including encapsulation technologies such as nanoemulsions, liposomes, or polymer-based delivery systems, could be employed to improve essential oil stability, protect bioactive compounds from degradation, and enable controlled release [90,91,92]. Future studies should therefore focus on optimizing such formulations and evaluating their stability, safety, and efficacy in more complex biological models.

## 5. Conclusions

This study demonstrated that biosynthesized AgNPs derived from *Lavandula angustifolia* and *Salvia rosmarinus*, in combination with their corresponding EOs and AMP, exhibit significant biological activity against a multidrug-resistant, biofilm-forming *S. equorum* strain. Despite the use of sub-MIC concentrations of all tested agents, the combined formulations consistently reduced bacterial growth and markedly inhibited biofilm formation. Although FICI analysis did not demonstrate a clear synergistic interaction, the observed predominantly additive effects resulted in biologically meaningful enhancement of antimicrobial activity compared with the individual components. Notably, the triple combinations were the only treatments capable of disrupting pre-established biofilms, highlighting the potential of this multi-component strategy against persistent biofilm-associated infections. Overall, these findings suggest that combining biosynthesized AgNPs, EOs, and AMP represents a promising approach for enhancing the efficacy of conventional antibiotic therapy against resistant biofilm-forming bacteria. Nevertheless, further studies are required to elucidate the underlying mechanisms of interaction, evaluate cytotoxicity, and validate the efficacy of this approach in relevant in vivo models.

## Figures and Tables

**Table 1:** Strain characterization.

Strain	Molecule Type	Isolation Source	Host	Length	Accession Number	Geographic Location Name	Collection Date
*S. equorum*	Genomic DNA	Skin	Dog	1419 bp	PV688033	Slovakia	25 February 2025

**Table 2:** Chemical composition of LEO according to the manufacturer’s data sheet.

Chemical Composition	Specification	Result
Linalool	20–50	43 ± 2
Linalyl acetate	–	39 ± 2

The values presented in the tables are expressed as percentages (%).

**Table 3:** Chemical composition of REO according to the manufacturer’s data sheet.

Chemical Composition	Specification	Result
α-pinene	18–26	19 ± 1
Camphene	8–12	9 ± 1
β-pinene	2–6	5 ± 1
β-myrcene	1.5–5	-
Limonene	2.5–5	-
Cineole	15–25	25 ± 1
*p*-cymene	1–2.2	17 ± 1
Camphor	13–21	19 ± 1
Bornylacetate	0.5–2.5	up to 2
α-terpineole	1–3.5	2.5 ± 0.2
Borneole	2–4.5	2 ± 0.5
Verbenone	0.7–2.5	-

The values presented in the tables are expressed as percentages (%).

**Table 4:** Overall biological activity of AgNPs-L, LEO, and their combinations with AMP against the resistant and biofilm-forming *S. equorum* strain.

Component	MIC	MBIC	MBEC
AgNPs-L	25	12.5	-
LEO	901	0.901	-
AgNPs-L/LEO	12.5/0.901	12.5/0.901	12.5/0.901
AgNPs-L/LEO/AMP	12.5/0.901/0.5	12.5/0.901/0.5	12.5/0.901/0.5
AgNPs-L/LEO/AMP	12.5/0.901/0.25	12.5/0.901/0.25	12.5/0.901/0.25

MIC = minimum inhibitory concentration; MBIC = minimum biofilm inhibitory concentration; MBEC = minimum biofilm eradication concentration; AgNPs-L = lavender-derived silver nanoparticles; LEO = lavender essential oil; AMP = ampicillin. Values are expressed in μg/mL.

**Table 5:** Overall biological activity of AgNPs-R, REO, and their combinations with AMP against the resistant and biofilm-forming *S. equorum* strain.

Component	MIC	MBIC	MBEC
AgNPs-R	12.5	6.25	-
REO	929	0.929	-
AgNPs-R/REO	6.25/0.929	6.25/0.929	6.25/0.929
AgNPs-R/REO/AMP 0.5 mg/L	6.25/0.929/0.5	6.25/0.929/0.5	6.25/0.929/0.5
AgNPs-R/REO/AMP 0.25 mg/L	6.25/0.929/0.25	6.25/0.929/0.25	6.25/0.929/0.25

MIC = minimum inhibitory concentration; MBIC = minimum biofilm inhibitory concentration; MBEC = minimum biofilm eradication concentration; AgNPs-R = rosemary-derived silver nanoparticles; REO = rosemary essential oil; AMP = ampicillin. Values are expressed in μg/mL.

## Data Availability

The original contributions presented in this study are included in the article. Further inquiries can be directed to the corresponding author.

## Supplementary Materials

The following supporting information can be downloaded at https://www.mdpi.com/article/10.3390/pharmaceutics18081017/s1. Figure S1: Dynamic light scattering (DLS) intensity size distribution of biosynthesized silver nanoparticles prepared using *Lavandula angustifolia* extract (AgNPs-L); Figure S2: Dynamic light scattering (DLS) intensity size distribution of biosynthesized silver nanoparticles prepared using *Salvia rosmarinus* extract (AgNPs-R); Figure S3: Zeta potential distribution of biosynthesized silver nanoparticles prepared using *Lavandula angustifolia* extract (AgNPs-L); Figure S4: Zeta potential distribution of biosynthesized silver nanoparticles prepared using *Salvia rosmarinus* extract (AgNPs-R); Figure S5: FTIR spectra of *Lavandula angustifolia* and *Salvia rosmarinus* extracts before and after the biosynthesis of silver nanoparticles (AgNPs); Figure S6: RP-HPLC-DAD chromatographic profile of the aqueous extract of Lavandula angustifolia recorded at λ = 280 nm (A) and λ = 330 nm (B); Figure S7: RP-HPLC-DAD chromatographic profile of the aqueous extract of Salvia rosmarinus recorded at λ = 280 nm (A) and λ = 330 nm (B); Table S1: Statistical analysis of the antibacterial activity of AgNPs; Table S2: Statistical analysis of the antibacterial activity of Eos; Table S3: Statistical analysis of the antibacterial activity of EOs, AgNPs and AMP combination; Table S4: Statistical analysis of the antibiofilm activity of AgNPs; Table S5: Statistical analysis of the antibiofilm activity of Eos; Table S6: Statistical analysis of the antibiofilm activity of EOs, AgNPs and AMP combination; Table S7: Statistical analysis of the biofilm eradication effect of AgNPs; Table S8: Statistical analysis of the biofilm eradication effect of Eos; Table S9: Statistical analysis of the biofilm eradication effect of EOs, AgNPs and AMP combination; Table S10: Phytochemical profile, retention times, molar concentrations, and relative abundances of quantified phenolic compounds in the aqueous extract of Lavandula angustifolia; Table S11: Phytochemical profile, retention times, molar concentrations, and relative abundances of quantified phenolic compounds in the aqueous extract of Salvia rosmarinus. References [93,94,95] are cited in the supplementary materials.

## Abbreviations

The following abbreviations are used in this manuscript:

AgNPs	silver nanoparticles
AgNPs-L	lavender-derived silver nanoparticles
AgNPs-R	rosemary-derived silver nanoparticles
AgNO_3_	silver nitrate
AMP	Ampicillin
AMR	antimicrobial resistance
ANOVA	analysis of variance
CLSI	Clinical and Laboratory Standards Institute
DLS	dynamic light scattering
EDS	energy-dispersive X-ray spectroscopy
ESKAPE	*Enterococcus faecium*, *Staphylococcus aureus*, *Klebsiella pneumoniae*, *Acinetobacter baumannii*, *Pseudomonas aeruginosa*, *Enterobacter* species—acronym
EOs	essential oils
EPS	extracellular polymeric substances
EUCAST	European Committee on Antimicrobial Susceptibility Testing
FIC	fractional inhibitory concentration
FICI	fractional inhibitory concentration index
FTIR	Fourier-transform infrared spectroscopy
GC/MS	gas chromatography/mass spectrometry
LEO	lavender essential oil
mBHI	modified brain heart infusion broth
MBEC	minimum biofilm eradication concentration
MBIC	minimum biofilm inhibitory concentration
MDR	multidrug resistance
MIC	minimum inhibitory concentration
MRSA	methicillin-resistant *Staphylococcus aureus*
NaCl	sodium chloride
NASM	non-*aureus* staphylococci and mammaliicocci
REO	rosemary essential oil
ROS	reactive oxygen species
SD	standard deviation
SEM	scanning electron microscopy
SPR	surface plasmon resonance
sub-MIC	sub-minimum inhibitory concentration
TEM	transmission electron microscopy
UV-Vis	ultraviolet–visible spectrophotometry
WHO	World Health Organization

## References

[1] Davies J., Davies D. (2010). Origins and evolution of antibiotic resistance. Microbiol. Mol. Biol. Rev..

[2] World Health Organization Antimicrobial Resistance. https://www.who.int/news-room/fact-sheets/detail/antimicrobial-resistance.

[3] von Wintersdorff C.J.H., Penders J., van Niekerk J.M., Mills N.D., Majumder S., van Alphen L.B., Savelkoul P.H.M., Wolffs P.F.G. (2016). Dissemination of Antimicrobial Resistance in Microbial Ecosystems through Horizontal Gene Transfer. Front. Microbiol..

[4] Liu H.Y., Prentice E.L., Webber M.A. (2024). Mechanisms of antimicrobial resistance in biofilms. npj Antimicrob. Resist..

[5] Alaoui Mdarhri H., Benmessaoud R., Yacoubi H., Seffar L., Guennouni Assimi H., Hamam M., Boussettine R., Filali-Ansari N., Lahlou F.A., Diawara I. (2022). Alternatives Therapeutic Approaches to Conventional Antibiotics: Advantages, Limitations and Potential Application in Medicine. Antibiotics.

[6] Asante J., Hetsa B.A., Amoako D.G., Abia A.L.K., Bester L.A., Essack S.Y. (2021). Multidrug-Resistant Coagulase-Negative Staphylococci Isolated from Bloodstream in the uMgungundlovu District of KwaZulu-Natal Province in South Africa: Emerging Pathogens. Antibiotics.

[7] Király J., Gregová G., Hudecová P., Hajdučková V., Hisirová S., Dančová N., Takáč P., Verebová V., Bedlovičová Z. (2026). Effect of Biosynthesized Gold and Silver Nanoparticles Using *Alchemilla vulgaris* Extract and Their Synergistic Action with Subinhibitory Concentrations of Ampicillin Against Staphylococci. Antibiotics.

[8] Even S., Leroy S., Charlier C., Zakour N.B., Chacornac J.-P., Lebert I., Jamet E., Desmonts M.-H., Coton E., Pochet S. (2010). Low occurrence of safety hazards in coagulase negative staphylococci isolated from fermented foodstuffs. Int. J. Food Microbiol..

[9] Vázquez L., Srednik M.E., Rodríguez J., Flórez A.B., Mayo B. (2023). Antibiotic Resistance/Susceptibility Profiles of *Staphylococcus equorum* Strains from Cheese, and Genome Analysis for Antibiotic Resistance Genes. Int. J. Mol. Sci..

[10] Michael C.K., Lianou D.T., Tsilipounidaki K., Gougoulis D.A., Giannoulis T., Vasileiou N.G.C., Mavrogianni V.S., Petinaki E., Fthenakis G.C. (2023). Recovery of Staphylococci from Teatcups in Milking Parlours in Goat Herds in Greece: Prevalence, Identification, Biofilm Formation, Patterns of Antibiotic Susceptibility, Predictors for Isolation. Antibiotics.

[11] Chegini Z., Shariati A., Alikhani M.Y., Safaiee M., Rajaeih S., Arabestani M., Azizi M. (2024). Antibacterial and antibiofilm activity of silver nanoparticles stabilized with C-phycocyanin against drug-resistant *Pseudomonas aeruginosa* and *Staphylococcus aureus*. Front. Bioeng. Biotechnol..

[12] Jiang X., Khan S., Dykes A., Stulz E., Zhang X. (2025). Biogenic Synthesis of Silver Nanoparticles and Their Diverse Biomedical Applications. Molecules.

[13] Munir S., Amanat T., Raja M.A., Mohammed K., Rasheed R.A., Hussein D.S., Shireen F., Ahmad J., Ahmad Z., Hayat S. (2023). Antimicrobial efficacy of phyto-synthesized silver nanoparticles using aqueous leaves extract of *Rosamarinus officinalis* L. Pak. J. Pharm. Sci..

[14] Madhusudanan M., Mijakovic I., Singh P. (2026). Biogenic vs. Chemical AgNPs: A Comparison of Antimicrobial Potency and Stability. Int. J. Mol. Sci..

[15] Franci G., Falanga A., Galdiero S., Palomba L., Rai M., Morelli G., Galdiero M. (2015). Silver Nanoparticles as Potential Antibacterial Agents. Molecules.

[16] Hudecová P., Koščová J., Hajdučková V., Király J., Horňak P. (2024). Antibacterial and Antibiofilm Activity of Essential Oils Against *Aeromonas* spp. Isolated from Rainbow Trout. Animals.

[17] Kajjari S., Joshi R.S., Hugar S.M., Gokhale N., Meharwade P., Uppin C. (2022). The Effects of Lavender Essential Oil and its Clinical Implications in Dentistry: A Review. Int. J. Clin. Pediatr. Dent..

[18] Rahbardar M.G., Hosseinzadeh H. (2020). Therapeutic effects of rosemary (*Rosmarinus officinalis* L.) and its active constituents on nervous system disorders. Iran. J. Basic. Med. Sci..

[19] Xuan L., Ju Z., Skonieczna M., Zhou P.-K., Huang R. (2023). Nanoparticles-induced potential toxicity on human health: Applications, toxicity mechanisms, and evaluation models. MedComm.

[20] El-Gebaly A.S., Sofy A.R., Hmed A.A., Youssef A.M. (2024). Combination of nanoparticles (NPs) and essential oils (EOs) as promising alternatives to non-effective antibacterial, antifungal and antiviral agents: A review. Biocatal. Agric. Biotechnol..

[21] Hisirová S., Koščová J., Király J., Hajdučková V., Hudecová P., Lauko S., Gregová G., Dančová N., Koreneková J., Lovayová V. (2025). Resistance Genes and Virulence Factor Genes in Coagulase-Negative and Positive Staphylococci of the *Staphylococcus intermedius* Group (SIG) Isolated from the Dog Skin. Microorganisms.

[22] Mustapha T., Misni N., Ithnin N.R., Daskum A.M., Unyah N.Z. (2022). A Review on Plants and Microorganisms Mediated Synthesis of Silver Nanoparticles, Role of Plants Metabolites and Applications. Int. J. Environ. Res. Public Health.

[23] Thatyana M., Dube N.P., Kemboi D., Manicum A.-L.E., Mokgalaka-Fleischmann N.S., Tembu J.V. (2023). Advances in Phytonanotechnology: A Plant-Mediated Green Synthesis of Metal Nanoparticles Using *Phyllanthus* Plant Extracts and Their Antimicrobial and Anticancer Applications. Nanomaterials.

[24] Mittal A.K., Chisti Y., Banerjee U.C. (2013). Synthesis of metallic nanoparticles using plant extracts. Biotechnol. Adv..

[25] O’Toole G.A., Pratt L.A., Watnick P.I., Newman D.K., Weaver V.B.K.R. (1999). Genetic approaches to study of biofilms. Methods Enzymol..

[26] Ayubee M.S., Akter F., Ahmed N.T., Kabir A.K.L., Hossain M.M., Hussain M.D., Kazi M., Mazid M.A. (2025). Synergistic antibacterial action of AgNP-ampicillin conjugates: Evading β-lactamase degradation in ampicillin-resistant clinical isolates. PLoS ONE.

[27] Kelly K.L., Coronado E., Zhao L.L., Schatz G.C. (2003). The optical properties of metal nanoparticles: The influence of size, shape, and dielectric environment. J. Phys. Chem..

[28] Li K., Geng T., Wang S., Li H., Hu N., Zhang H., You L., Zhou C., Liz-Marzán L.M., Ni W. (2026). Plasmon damping induced by ferroelectric phase transition in a single plasmonic nanocavity. ACS Photonics.

[29] Asif M., Yasmin R., Asif R., Ambreen A., Mustafa M., Umbreen S. (2022). Green synthesis of silver nanoparticles (AgNPs), structural characterization, and their antibacterial potential. Dose-Response.

[30] Rai M., Yadav A., Gade A. (2009). Silver nanoparticles as a new generation of antimicrobials. Biotechnol. Adv..

[31] Singh P., Kim Y.J., Zhang D., Yang D.C. (2016). Biological synthesis of nanoparticles from plants and microorganisms. Trends Biotechnol..

[32] Khan M., Al-Hamoud K., Liaqat Z., Shaik M.R., Adil S.F., Kuniyil M., Alkhathlan H.A., Al-Warthan A., Siddiqui M.R.H., Mondeshki M. (2020). Synthesis of Au, Ag, and Au–Ag bimetallic nanoparticles using Pulicaria undulata extract and their catalytic activity for the reduction of 4-nitrophenol. Nanomaterials.

[33] Sellami H., Khan S.A., Ahmad I., Alarfaj A.A., Hirad A.H., Al-Sabri A.E. (2021). Green synthesis of silver nanoparticles using Olea europaea leaf extract for their enhanced antibacterial, antioxidant, cytotoxic and biocompatibility applications. Int. J. Mol. Sci..

[34] Yakoup A.Y., Kamel A.G., Elbermawy Y., Abdelsattar A.S., El-Shibiny A. (2024). Characterization, antibacterial, and cytotoxic activities of silver nanoparticles using the whole biofilm layer as a macromolecule in biosynthesis. Sci. Rep..

[35] Ahsan A., Farooq M.A., Ahsan Bajwa A., Parveen A. (2020). Green Synthesis of Silver Nanoparticles Using Parthenium Hysterophorus: Optimization, Characterization and In Vitro Therapeutic Evaluation. Molecules.

[36] Hashemi Z., Shirzadi-Ahodashti M., Mortazavi-Derazkola S., Ebrahimzadeh M.A. (2022). Sustainable biosynthesis of metallic silver nanoparticles using barberry phenolic extract: Optimization and evaluation of photocatalytic, in vitro cytotoxicity, and antibacterial activities against multidrug-resistant bacteria. Inorg. Chem. Commun..

[37] Santos M.H.d.M., Endo T.H., Scandorieiro S., Pavanelli W.R., Kobayashi R.K.T., Nakazato G. (2025). Silver Nanoparticle–Antibiotic Combinations: A Strategy to Overcome Bacterial Resistance in *Escherichia coli*, *Salmonella* Enteritidis and *Staphylococcus aureus*. Antibiotics.

[38] Onem E., Sivri F.M., Soyocak A., Ak A., Erzurumlu Y., Saribas R. (2026). Eco-Friendly Synthesis of Silver and Selenium Nanoparticles Using Lavandula angustifolia Drujba Extract and Evaluation of Their Antibacterial and Cytotoxic Properties. ChemistrySelect.

[39] Fadel B.A., Elwakil B.H., Fawzy E.E., Shaaban M.M., Olama Z.A. (2023). Nanoemulsion of *Lavandula angustifolia* Essential Oil/Gold Nanoparticles: Antibacterial Effect against Multidrug-Resistant Wound-Causing Bacteria. Molecules.

[40] Ödemiş Ö., Özdemir S., Gonca S., Ağırtaş M.S. (2024). Characterization of silver nanoparticles fabricated by green synthesis using *Urtica dioica* and *Lavandula angustifolia* and investigation of antimicrobial and antioxidant. Inorg. Nano-Metal. Chem..

[41] Illanes Tormena R.P., Medeiros Salviano Santos M.K., Oliveira da Silva A., Félix F.M., Chaker J.A., Freire D.O., Rodrigues da Silva I.C., Moya S.E., Sousa M.H. (2024). Enhancing the antimicrobial activity of silver nanoparticles against pathogenic bacteria by using *Pelargonium sidoides* DC extract in microwave assisted green synthesis. RSC Adv..

[42] Khaleel N.M.A., Sarmamz A.O.I. (2022). Antibacterial effects of the extracts of rosemary (*Rosmarinus officinalis* L.) Leaves on three multidrug resistant bacterial isolates using nanoparticle size technique. J. Pharm. Negat. Results.

[43] Mačák L., Velgosová O., Múdra E., Vojtko M., Ondrašovičová S. (2026). Sustainable Lavender Extract-Mediated Synthesis of Silver Nanoparticles and Their Use in Fabricating Antibacterial Polymer Nanocomposites. Nanomaterials.

[44] Elmehalawy N.G., Zaky M.M.M., Eid A.M., Fouda A. (2025). Eco-friendly synthesis of silver nanoparticles: Multifaceted antioxidant, antidiabetic, anticancer, and antimicrobial activities. Sci. Rep..

[45] Al-Audah S.A., Alghamdi A.I., Alsanie S.I., Alabdalla N.M., Alawdah A., Alenezi N., AlShammari A., Taha I., Albarrag A., Aldakeel S. (2026). Green Synthesis of Silver Nanoparticles with Antibacterial, Anti-Inflammatory, and Antioxidant Activity Using *Convolvulus arvensis*. Int. J. Mol. Sci..

[46] Yusuf-Omoloye N.A., Adeyemi F.M., Sule W.F., Azeez L., Oyedara O.O., Wahab A.A., Ajigbewu O.H., Lateef A. (2024). Green-synthesis, characterization, and antibacterial activity of *Azanza garckeana* seed extract silver nanoparticles against vancomycin-resistant *Enterococci*. Next Nanotechnol..

[47] Sharif I.H., Primu F.S., Joy M.N.H., Tithi M.H., Chowdhury A., Pretha P.D., Jamal A.H.M., Roy T.K., Ismail Z., Islam M.S. (2026). Green synthesis of silver nanoparticles from *Eichhornia crassipes* and evaluates their antimicrobial properties against multidrug-resistant UTI pathogens. Sci. Rep..

[48] Șuică-Bunghez I.R., Senin R.M., Sorescu A.A., Ganciarov M., Răut I., Firincă C., Constantin M., Gifu I.C., Stoica R., Fierăscu I. (2024). Application of *Lavandula angustifolia* Mill. Extracts for the Phytosynthesis of Silver Nanoparticles: Characterization and Biomedical Potential. Plants.

[49] Akdaşçi E., Eker F., Duman H., Bechelany M., Karav S. (2025). Microbial-Based Green Synthesis of Silver Nanoparticles: A Comparative Review of Bacteria- and Fungi-Mediated Approaches. Int. J. Mol. Sci..

[50] Swidan N.S., Hashem Y.A., Elkhatib W.F., Yassien M.A. (2022). Antibiofilm activity of green synthesized silver nanoparticles against biofilm associated enterococcal urinary pathogens. Sci. Rep..

[51] Jaiswal S., Mishra P. (2018). Antimicrobial and antibiofilm activity of curcumin-silver nanoparticles with improved stability and selective toxicity to bacteria over mammalian cells. Med. Microbiol. Immunol..

[52] Hamid M.T., Hussein N.N., Sulaiman G.M., Mohammed H.A. (2025). Antibacterial and antibiofilm properties of silver nanoparticles synthesized using *Carthamus tinctorius* extract against various multidrug-resistant bacterial strains. Discov. Appl. Sci..

[53] Gopinath K., Kumaraguru S., Bhakyaraj K., Mohan S., Venkatesh K.S., Esakkirajan M., Kaleeswarran P., Alharbi N.S., Kadaikunnan S., Govindarajan M. (2016). Green synthesis of silver, gold and silver/gold bimetallic nanoparticles using the *Gloriosa superba* leaf extract and their antibacterial and antibiofilm activities. Microb. Pathog..

[54] Hosnedlova B., Kabanov D., Kepinska M., Narayanan V.H.B., Parikesit A.A., Fernandez C., Bjørklund G., Nguyen H.V., Farid A., Sochor J. (2022). Effect of Biosynthesized Silver Nanoparticles on Bacterial Biofilm Changes in *S. aureus* and *E. coli*. Nanomaterials.

[55] Wahab S., Asmare M.M., Khan A., Khan T., Ahmad R., Sunghwan K., Yun S.-I. (2025). Green synthesis of silver nanoparticles using *salvia rosmarinus* extract: Characterization, mechanistic antibacterial, antibiofilm, and in silico evaluation. Ind. Crops Prod..

[56] Raj S., Trivedi R., Soni V. (2022). Biogenic Synthesis of Silver Nanoparticles, Characterization and Their Applications—A Review. Surfaces.

[57] Kavanaugh N.L., Ribbeck K. (2012). Selected antimicrobial essential oils eradicate *Pseudomonas* spp. and *Staphylococcus aureus* biofilms. Appl. Environ. Microbiol..

[58] Bakkali F., Averbeck S., Averbeck D., Idaomar M. (2008). Biological effects of essential oils—A review. Food Chem. Toxicol..

[59] Wińska K., Mączka W., Łyczko J., Grabarczyk M., Czubaszek A., Szumny A. (2019). Essential Oils as Antimicrobial Agents—Myth or Real Alternative?. Molecules.

[60] Liu T., Wang J., Gong X., Wu X., Liu L., Chi F. (2020). Rosemary and Tea Tree Essential Oils Exert Antibiofilm Activities In Vitro against *Staphylococcus aureus* and *Escherichia coli*. J. Food Prot..

[61] Kabotso D.E.K., Neglo D., Gaba S.E., Danyo E.K., Dayie A.D., Asantewaa A.A., Kotey F.C.N., Dayie N.T.K.D. (2024). In Vitro Evaluation of Rosemary Essential Oil: GC-MS Profiling, Antibacterial Synergy, and Biofilm Inhibition. Pharmaceuticals.

[62] Romo-Castillo M., Flores-Bautista V.A., Guzmán-Gutiérrez S.L., Reyes-Chilpa R., León-Santiago M., Luna-Pineda V.M. (2023). Synergy of Plant Essential Oils in Antibiotic Therapy to Combat *Klebsiella pneumoniae* Infections. Pharmaceuticals.

[63] Khalil D.Y., Hassan O.M. (2024). Antioxidant and Antibacterial Properties of Rosmarinus officinalis Essential Oil. J. Univ. Anbar Pure Sci..

[64] Cintra T.M.F., de Menezes R.T., de Carvalho L.S., de Miguel Nazario L., Hantao L.W., Marcucci M.C., de Oliveira L.D., Meccatti-Domiciano V.M. (2025). Rosemary Extract: Phytochemical Composition and Potential for Eliminating Polymicrobial Biofilm of *Candida albicans* and Multidrug-Resistant Bacteria. BioTech.

[65] Aniba R., Ramzi A., Dihmane A., Raqraq H., Ressmi A., Nayme K., Timinouni M., Mohammed E.I., Abdellah F., Barguigua A. (2025). Antimicrobial and anti-biofilm activities of the essential oils of *Lavandula angustifolia* Mill, *Salvia sclarea* L, and *Mentha pulegium* L against uropathogenic *Staphylococcus aureus* in vitro and in silico. S. Afr. J. Bot..

[66] Ben Abdallah F., Lagha R., Gaber A. (2020). Biofilm Inhibition and Eradication Properties of Medicinal Plant Essential Oils against Methicillin-Resistant *Staphylococcus aureus* Clinical Isolates. Pharmaceuticals.

[67] Bowbe K.H., Salah K.B.H., Moumni S., Ashkan M.F., Merghni A. (2023). Anti-Staphylococcal Activities of *Rosmarinus officinalis* and *Myrtus communis* Essential Oils through ROS-Mediated Oxidative Stress. Antibiotics.

[68] Stamova S., Ermenlieva N., Tsankova G., Georgieva E. (2025). Antimicrobial Activity of Lavender Essential Oil from *Lavandula angustifolia* Mill.: In Vitro and In Silico Evaluation. Antibiotics.

[69] Hossain S., Heo H., De Silva B.C.J., Wimalasena S.H.M.P., Pathirana H.N.K.S., Heo G.-J. (2017). Antibacterial activity of essential oil from lavender (*Lavandula angustifolia*) against pet turtle-borne pathogenic bacteria. Lab. Anim. Res..

[70] Brożyna M., Paleczny J., Kozłowska W., Chodaczek G., Dudek-Wicher R., Felińczak A., Gołębiewska J., Górniak A., Junka A. (2021). The Antimicrobial and Antibiofilm In Vitro Activity of Liquid and Vapour Phases of Selected Essential Oils against *Staphylococcus aureus*. Pathogens.

[71] Cui Z.-H., He H.-L., Wu S.-B., Dong C.-L., Lu S.-Y., Shan T.-J., Fang L.-X., Liao X.-P., Liu Y.-H., Sun J. (2021). Rapid Screening of Essential Oils as Substances Which Enhance Antibiotic Activity Using a Modified Well Diffusion Method. Antibiotics.

[72] Truong S., Mudgil P. (2023). The antibacterial effectiveness of lavender essential oil against methicillin-resistant *Staphylococcus aureus*: A systematic review. Front. Pharmacol..

[73] Vanegas D., Abril-Novillo A., Khachatryan A., Jerves-Andrade L., Peñaherrera E., Cuzco N., Wilches I., Calle J., León-Tamariz F. (2021). Validation of a method of broth microdilution for the determination of antibacterial activity of essential oils. BMC Res. Notes..

[74] Spadini C., Iannarelli M., Carrillo Heredero A.M., Montanaro S.L., Mezzasalma N., Simoni M., Righi F., Cabassi C.S. (2024). Stability of the antimicrobial activity of selected essential oils and nature identical compounds and their interaction with Tween 20 against reference bacterial strains of zootechnical interest. Ital. J. Anim. Sci..

[75] Dávila-Rodríguez M., López-Malo A., Palou E., Ramírez-Corona N., Jiménez-Munguía M.T. (2020). Essential oils microemulsions prepared with high-frequency ultrasound: Physical properties and antimicrobial activity. J. Food Sci. Technol..

[76] Walasek-Janusz M., Grzegorczyk A., Zalewski D., Malm A., Gajcy S., Gruszecki R. (2022). Variation in the Antimicrobial Activity of Essential Oils from Cultivars of *Lavandula angustifolia* and *L.* × *intermedia*. Agronomy.

[77] Jeong D.W., Han S., Lee J.H. (2014). Safety and technological characterization of *Staphylococcus equorum* isolates from jeotgal, a Korean high-salt-fermented seafood, for starter development. Int. J. Food Microbiol..

[78] Dakal T.C., Kumar A., Majumdar R.S., Yadav V. (2016). Mechanistic Basis of Antimicrobial Actions of Silver Nanoparticles. Front. Microbiol..

[79] Murei A., Ayinde W.B., Gitari M.W., Samie A. (2020). Functionalization and antimicrobial evaluation of ampicillin, penicillin and vancomycin with *Pyrenacantha grandiflora* Baill and silver nanoparticles. Sci. Rep..

[80] Masadeh M.M., Al-Tai Z., Khanfar M.S., Alzoubi K.H., Sabi S.H., Masadeh M.M. (2024). Synergistic Effect of Silver Nanoparticles with Antibiotics for Eradication of Pathogenic Biofilms. Curr. Pharm. Biotechnol..

[81] Kadi R.H.H. (2025). Synergistic Effects of Silver Nanoparticles Combined with Amoxicillin or Cymbopogon schoenanthus Essential Oil on the Formation of Bacterial Biofilm. J. Contemp. Med. Sci..

[82] Meroni G., Laterza G., Tsikopoulos A., Tsikopoulos K., Vitalini S., Scaglia B., Iriti M., Bonizzi L., Martino P.A., Soggiu A. (2024). Antibacterial Potential of Essential Oils and Silver Nanoparticles against Multidrug-Resistant *Staphylococcus pseudintermedius* Isolates. Pathogens.

[83] Chouhan S., Sharma K., Guleria S. (2017). Antimicrobial Activity of Some Essential Oils—Present Status and Future Perspectives. Medicines.

[84] Scandorieiro S., Teixeira F.M.M.B., Nogueira M.C.L., Panagio L.A., de Oliveira A.G., Durán N., Nakazato G., Kobayashi R.K.T. (2023). Antibiofilm Effect of Biogenic Silver Nanoparticles Combined with Oregano Derivatives against Carbapenem-Resistant *Klebsiella pneumoniae*. Antibiotics.

[85] Heo S., Oh S.-E., Lee G., Lee J., Ha N.-C., Jeon C.O., Jeong K., Lee J.-H., Jeong D.-W. (2023). *Staphylococcus equorum* plasmid pKS1030-3 encodes auxiliary biofilm formation and trans-acting gene mobilization systems. Sci. Rep..

[86] Casals E., Gusta M.F., Bastus N., Rello J., Puntes V. (2025). Silver Nanoparticles and Antibiotics: A Promising Synergistic Approach to Multidrug-Resistant Infections. Microorganisms.

[87] Sivasankaran A., Jonnalagadda S. (2025). Nano-Based Antibiofilm Strategy Against *Pseudomonas aeruginosa*. Biosci. Biotechnol. Res. Asia.

[88] Nuță D.C., Limban C., Chiriță C., Chifiriuc M.C., Costea T., Ioniță P., Nicolau I., Zarafu I. (2021). Contribution of Essential Oils to the Fight against Microbial Biofilms—A Review. Processes.

[89] Turek C., Stintzing F.C. (2013). Stability of essential oils: A review. Compr. Rev. Food Sci. Food Saf..

[90] Sherry M., Charcosset C., Fessi H., Greige-Gerges H. (2013). Essential oils encapsulated in liposomes: A review. J. Liposome Res..

[91] Lim X.-Y., Li J., Yin H.-M., He M., Li L., Zhang T. (2023). Stabilization of Essential Oil: Polysaccharide-Based Drug Delivery System with Plant-like Structure Based on Biomimetic Concept. Polymers.

[92] de Almeida D.P., Azevedo P.Z., Rodrigues R.C., Lima E.d.P.R., Stringueta P.C., Campelo P.H., Martins E. (2026). Nanoemulsion-Based Delivery of Essential Oils for Controlling Foodborne Pathogens and Spoilage Microorganisms. Micro.

[93] Oalđe Pavlović M., Milutinović M., Alimpić Aradski A., Gašić U., Mišić D., Marin P.D., Duletić-Laušević S. (2026). Therapeutic Potential of Salvia rosmarinus: Seasonal and Geographical Variation in Phytochemical Composition, Bioactivity, and Synergistic Effects of Rosmarinic Acid with 5-FU. Plants.

[94] Musolino V., Macrì R., Cardamone A., Tucci L., Serra M., Lupia C., Maurotti S., Mare R., Nucera S., Guarnieri L. (2023). Salvia rosmarinus Spenn. (Lamiaceae) Hydroalcoholic Extract: Phytochemical Analysis, Antioxidant Activity and In Vitro Evaluation of Fatty Acid Accumulation. Plants.

[95] Dhouibi N., Manuguerra S., Arena R., Messina C.M., Santulli A., Kacem S., Dhaouadi H., Mahdhi A. (2023). Impact of the Extraction Method on the Chemical Composition and Antioxidant Potency of Rosmarinus officinalis L. Extracts. Metabolites.

