# Supplementary material for: Exploitation of Nanoparticle–Essential Oil Combinations to Enhance the Efficacy of Antimicrobial Agents Against Staphylococcus equorum
Source: Pharmaceutics. 2026 Aug 17;18(8):1017. doi: 10.3390/pharmaceutics18081017 (PMC13516417; doi:10.3390/pharmaceutics18081017)
Supplement: Supplementary file 1 [file pharmaceutics-18-01017-s001.zip › pharmaceutics-4475838-supplementary.pdf]

Supplementary Tables S1–S9. Statistical analysis of the antibacterial, antibiofilm and biofilm eradication activities of AgNPs, LEO, REO and the combination of AgNPs, EOs and AMP

The supplementary tables summarize the statistical analyses corresponding to the data presented in Figures 6 – 14. Values are expressed as mean ± SD (n = 3), and statistical significance was evaluated using one-way ANOVA followed by Dunnett's test. Adjusted p-values represent comparisons between each tested concentration and the corresponding positive control.

Table S1. Statistical analysis of the antibacterial activity of AgNPs

Concentration of active substances	Mean ± SD	One-way ANOVA (F; p-value)	Dunnett´s test (adjusted p-value)
Antibacterial activity of AgNPs-L			
S. equorum **SD75** (positive control)	0.391 ± 0.008	F (5, 12) = 3251, p < 0.0001	-
100 µg/mL	0.030 ± 0.005		p < 0.0001
50 µg/mL	0.038 ± 0.005		p < 0.0001
25 µg/mL	0.133 ± 0.006		p < 0.0001
12.5 µg/mL	0.388 ± 0.006		p = 0.8748
6.25 µg/mL	0.389 ± 0.003		p = 0. 9598
Antibacterial activity of AgNPs-R			
***S. equorum* SD75**	0.393 ± 0.005	F (5, 12) = 3619, p < 0.0001	-
100 µg/mL	0.015 ± 0.003		p < 0.0001
50 µg/mL	0.052 ± 0.006		p < 0.0001
25 µg/mL	0.055 ± 0.005		p < 0.0001
12.5 µg/mL	0.140 ± 0.007		p < 0.0001
6.25 µg/mL	0.384 ± 0.004		p = 0.1376

Table S2. Statistical analysis of the antibacterial activity of EOs

Concentration of active substances	Mean ± SD	One-way ANOVA (F; p-value)	Dunnett´s test (adjusted p-value)
Antibacterial activity of LEO			
***S. equorum* SD75**	0.391 ± 0.008	F (5, 12) = 2665, p < 0.0001	-
1.0%	0.015 ± 0.001		p < 0.0001
0.1%	0.103 ± 0.003		p < 0.0001
0.01%	0.381 ± 0.009		p = 0.1632
0.001%	0.382 ± 0.006		p = 0.2548
0.0001%	0.390 ± 0.004		p = 0.9955
Antibacterial activity of REO			
***S. equorum* SD75**	0.399 ± 0.010	F (5, 12) = 964, p < 0.0001	-
1.0%	0.083 ± 0.009		p < 0.0001
0.1%	0.137 ± 0.002		p < 0.0001
0.01%	0.382 ± 0.006		p = 0.0993
0.001%	0.387 ± 0.013		p = 0.3260
0.0001%	0.396 ± 0.005		p = 0.9927

Table S3. Statistical analysis of the antibacterial activity of EOs, AgNPs and AMP combination

Concentration of active substances	Mean ± SD	One-way ANOVA (F; p-value)	Dunnett´s test (adjusted p-value)
Antibacterial activity of AgNPs-L, LEO, AMP			
***S. equorum* SD75**	0.391 ± 0.003	F (5, 12) = 2028, p < 0.0001	-
AgNPs 12.5 µg/mL + EO 0.01%	0.180 ± 0.004		p < 0.0001
AgNPs 12.5 µg/mL + EO 0.01% + AMP 0.5 mg/L	0.133 ± 0.002		p < 0.0001
AgNPs 12.5 µg/mL + EO 0.01% + AMP 0.25 mg/L	0.140 ± 0.004		p < 0.0001
S. equorum + AMP 0.5 mg/L	0.382 ± 0.008		p = 0.1646
S. equorum + AMP 0.25 mg/L	0.388 ± 0.007		p = 0.9438
Antibacterial activity of AgNPs-R, REO, AMP			
***S. equorum* SD75**	0.391 ± 0.003	F (5, 12) = 1275, p < 0.0001	-
AgNPs 6.25 µg/mL + EO 0.01%	0.236 ± 0.003		p < 0.0001
AgNPs 6.25 µg/mL + EO 0.01% + AMP 0.5 mg/L	0.169 ± 0.002		p < 0.0001
AgNPs 6.25 µg/mL + EO 0.01% + AMP 0.25 mg/L	0.189 ± 0.008		p < 0.0001
S. equorum + AMP 0.5 mg/L	0.385 ± 0.007		p = 0.4564
S. equorum + AMP 0.25 mg/L	0.388 ± 0.007		p = 0.9496

Table S4. Statistical analysis of the antibiofilm activity of AgNPs

Concentration of active substances	Mean ± SD	One-way ANOVA (F; p-value)	Dunnett´s test (adjusted p-value)
Antibiofilm activity of AgNPs-L			
***S. equorum* SD75**	1.287 ± 0.022	F (5, 12) = 2131, p < 0.0001	-
100 µg/mL	0.147 ± 0.003		p < 0.0001
50 µg/mL	0.163 ± 0.021		p < 0.0001
25 µg/mL	0.171 ± 0.012		p < 0.0001
12.5 µg/mL	0.488 ± 0.011		p < 0.0001
6.25 µg/mL	1.247 ± 0.036		p = 0. 1228
Antibiofilm activity of AgNPs-R			
***S. equorum* SD75**	1.287 ± 0.022	F (5, 12) = 3956, p < 0.0001	-
100 µg/mL	0.059 ± 0.007		p < 0.0001
50 µg/mL	0.092 ± 0.004		p < 0.0001
25 µg/mL	0.098 ± 0.007		p < 0.0001
12.5 µg/mL	0.154 ± 0.007		p < 0.0001
6.25 µg/mL	0.479 ± 0.020		p < 0.0001

Table S5. Statistical analysis of the antibiofilm activity of EOs

Concentration of active substances	Mean ± SD	One-way ANOVA (F; p-value)	Dunnett´s test (adjusted p-value)

Antibiofilm activity of LEO			
***S. equorum* SD75**	1.003 ± 0.005	F (5, 12) = 2654, p < 0.0001	-
1.0%	0.136 ± 0.011		p < 0.0001
0.1%	0.167 ± 0.010		p < 0.0001
0.01%	0.462 ± 0.022		p < 0.0001
0.001%	0.971 ± 0.021		p = 0.0597
0.0001%	0.983 ± 0.007		p = 0.3138
Antibiofilm activity of REO			
***S. equorum* SD75**	1.003 ± 0.005	F (5, 12) = 2997, p < 0.0001	-
1.0%	0.041 ± 0.004		p < 0.0001
0.1%	0.211 ± 0.014		p < 0.0001
0.01%	0.463 ± 0.028		p < 0.0001
0.001%	0.983 ± 0.005		p = 0.3120
0.0001%	0.990 ± 0.010		p = 0.6345

Table S6. Statistical analysis of the antibiofilm activity of EOs, AgNPs and AMP combination

Concentration of active substances	Mean ± SD	One-way ANOVA (F; p-value)	Dunnett´s test (adjusted p-value)
Antibiofilm activity of AgNPs-L, LEO, AMP			
***S. equorum* SD75**	1.193 ± 0.006	F (5, 12) = 10442, p < 0.0001	-
AgNPs 12.5 µg/mL + EO 0.01%	0.448 ± 0.007		p < 0.0001
AgNPs 12.5 µg/mL + EO 0.01% + AMP 0.5 mg/L	0.265 ± 0.010		p < 0.0001
AgNPs 12.5 µg/mL + EO 0.01% + AMP 0.25 mg/L	0.347 ± 0.011		p < 0.0001
S. equorum + AMP 0.5 mg/L	1.182 ± 0.007		p = 0.3402
S. equorum + AMP 0.25 mg/L	1.192 ± 0.004		p > 0.9999
Antibiofilm activity of AgNPs-R, REO, AMP			
***S. equorum* SD75**	1.193 ± 0.006	F (5, 12) = 2223, p < 0.0001	-
AgNPs 6.25 µg/mL + EO 0.01%	0.492 ± 0.006		p < 0.0001
AgNPs 6.25 µg/mL + EO 0.01% + AMP 0.5 mg/L	0.368 ± 0.025		p < 0.0001
AgNPs 6.25 µg/mL + EO 0.01% + AMP 0.25 mg/L	0.452 ± 0.025		p < 0.0001
S. equorum + AMP 0.5 mg/L	1.191 ± 0.010		p = 0.9999
S. equorum + AMP 0.25 mg/L	1.192 ± 0.004		p > 0.9999

Table S7. Statistical analysis of the biofilm eradication effect of AgNPs

Concentration of active substances	Mean ± SD	One-way ANOVA (F; p-value)	Dunnett´s test (adjusted p-value)
Eradication effect of AgNPs-L			
***S. equorum* SD75** (positive control)	2.004 ± 0.004	F (5, 12) = 0.4346, p = 0.8161	-
100 µg/mL	1.982 ± 0.029		p = 0.6665
50 µg/mL	1.996 ± 0.024		p = 0.9915
25 µg/mL	1.998 ± 0.025		p = 0.9961
12.5 µg/mL	2.004 ± 0.021		p > 0.9999

6.25 μg/mL	2.006 ± 0.030		p > 0.9999
Eradication effect of AgNPs-R			
***S. equorum* SD75**	2.004 ± 0.004	F (5, 12) = 2.331, p = 0.1066	-
100 μg/mL	1.955 ± 0.010		p = 0.1108
50 μg/mL	1.961 ± 0.043		p = 0.1851
25 μg/mL	1.963 ± 0.022		p = 0.2055
12.5 μg/mL	1.972 ± 0.020		p = 0.3944
6.25 μg/mL	2.003 ± 0.029		p > 0.9999

Table S8. Statistical analysis of the biofilm eradication effect of EOs

Concentration of active substances	Mean ± SD	One-way ANOVA (F; p-value)	Dunnett´s test (adjusted p-value)
Eradication effect of LEO			
***S. equorum* SD75**	2.098 ± 0.007	F (5, 12) = 1.268, p = 0.3391	-
1.0%	2.064 ± 0.042		p = 0.2210
0.1%	2.081 ± 0.022		p = 0.7766
0.01%	2.085 ± 0.010		p = 0.8859
0.001%	2.097 ± 0.008		p > 0.9999
0.0001%	2.098 ± 0.009		p > 0.9999
Eradication effect of REO			
***S. equorum* SD75**	2.098 ± 0.007	F (5, 12) = 0.4059, p = 0.8356	-
1.0%	2.086 ± 0.006		p = 0.6651
0.1%	2.094 ± 0.006		p = 0.9954
0.01%	2.096 ± 0.024		p = 0.9997
0.001%	2.098 ± 0.014		p > 0.9999
0.0001%	2.098 ± 0.008		p > 0.9999

Table S9. Statistical analysis of the biofilm eradication effect of EOs, AgNPs and AMP combination

Concentration of active substances	Mean ± SD	One-way ANOVA (F; p-value)	Dunnett´s test (adjusted p-value)
Eradication effect of AgNPs-L, LEO, AMP			
***S. equorum* SD75**	1.682 ± 0.008	F (5, 12) = 2587, p < 0.0001	-
AgNPs 12.5 μg/mL + EO 0.01%	1.527 ± 0.001		p < 0.0001
AgNPs 12.5 μg/mL + EO 0.01% + AMP 0.5 mg/L	1.087 ± 0.015		p < 0.0001
AgNPs 12.5 μg/mL + EO 0.01% + AMP 0.25 mg/L	1.154 ± 0.005		p < 0.0001
S. equorum + AMP 0.5 mg/L	1.683 ± 0.013		p = 0.9999
S. equorum + AMP 0.25 mg/L	1.682 ± 0.008		p > 0.9999
Eradication effect of AgNPs-R, REO, AMP			
***S. equorum* SD75**	1.682 ± 0.008	F (5, 12) = 2359, p < 0.0001	-
AgNPs 12.5 μg/mL + EO 0.01%	1.594 ± 0.009		p < 0.0001
AgNPs 12.5 μg/mL + EO 0.01% + AMP 0.5 mg/L	0.960 ± 0.008		p < 0.0001

AgNPs 12.5 μg/mL + EO 0.01% + AMP 0.25 mg/L	1.038 ± 0.018		$p < 0.0001$
S. equorum + AMP 0.5 mg/L	1.655 ± 0.018		$p = 0.0722$
S. equorum + AMP 0.25 mg/L	1.682 ± 0.005		$p > 0.9999$

Supplementary Material and Methods and Figures S1–S5

The supplementary material provides additional characterization of biosynthesized silver nanoparticles obtained using *Lavandula angustifolia* and *Salvia rosmarinus* extracts. The included DLS and zeta potential measurements provide information on nanoparticle size distribution and colloidal stability, while FTIR analysis supports the identification of plant-derived functional groups involved in silver ion reduction and nanoparticle stabilization.

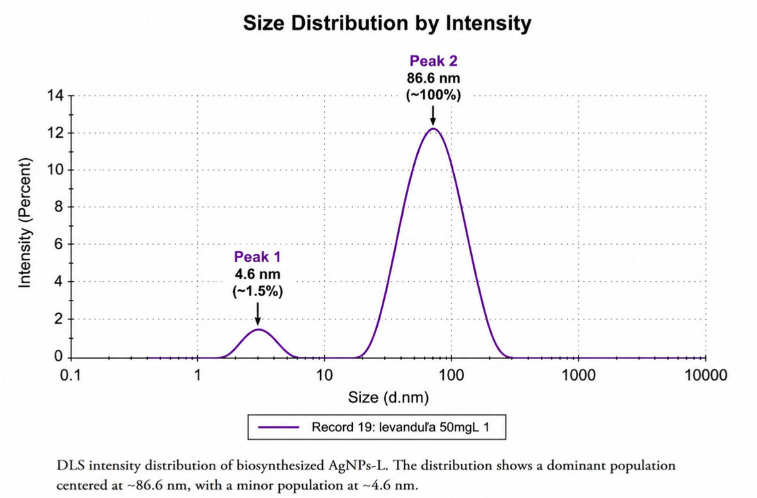

Figure S1. Dynamic light scattering (DLS) intensity size distribution of biosynthesized silver nanoparticles prepared using *Lavandula angustifolia* extract (AgNPs-L).

The intensity distribution revealed a dominant particle population with a hydrodynamic diameter centred at approximately **88.6 nm**, accompanied by a minor fraction of smaller particles around **4.6 nm**. The measurements were performed in triplicate, demonstrating good reproducibility of the particle size distribution.

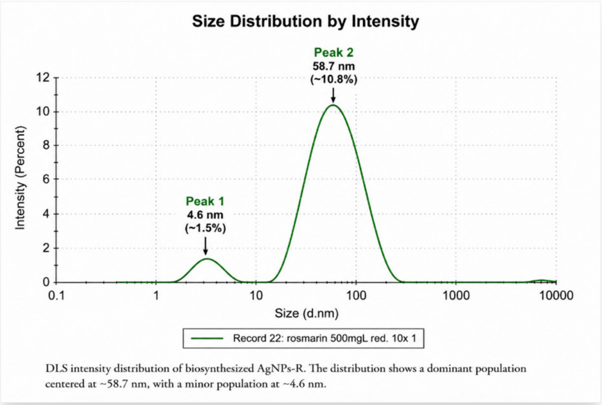

Figure S2. Dynamic light scattering (DLS) intensity size distribution of biosynthesized silver nanoparticles prepared using *Salvia rosmarinus* extract (AgNPs-R).

The intensity distribution showed a dominant particle population with a hydrodynamic diameter centred at approximately **58.7 nm**, together with a minor population of particles around **4.6 nm**. Triplicate measurements confirmed good reproducibility of the obtained size distribution.

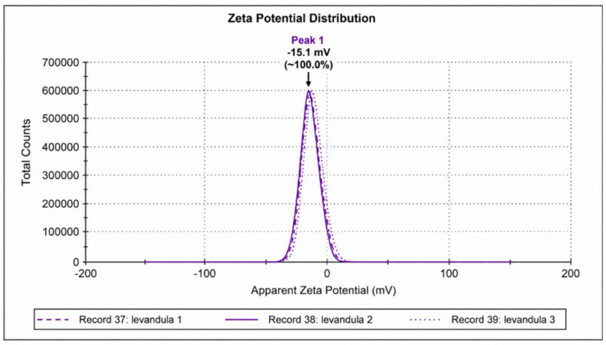

Figure S3. Zeta potential distribution of biosynthesized AgNPs-L (*Lavandula angustifolia* extract). The distribution shows a single dominant peak centered at −15.1 mV (~100.0%). Measurements were performed in triplicate, showing consistent results.

Figure S3. Zeta potential distribution of biosynthesized silver nanoparticles prepared using *Lavandula angustifolia* extract (AgNPs-L).

The zeta potential distribution exhibited a single dominant peak centred at **−15.1 mV**, indicating a homogeneous surface charge distribution of the synthesized nanoparticles. Triplicate measurements showed excellent agreement, confirming good repeatability and reproducibility of the zeta potential analysis.

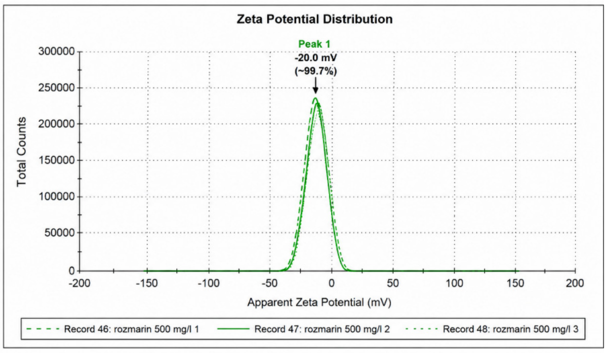

Figure S4. Zeta potential distribution of biosynthesized AgNPs-R (*Rosmarinus officinalis* extract). The distribution is characterized by a dominant peak centered at −20.0 mV (~99.7%). Measurements were performed in triplicate, showing consistent results.

Figure S4. Zeta potential distribution of biosynthesized silver nanoparticles prepared using *Salvia rosmarinus* extract (AgNPs-R).

The zeta potential distribution was characterized by a dominant peak centred at **−20.0 mV**, corresponding to the predominant nanoparticle population. Triplicate measurements demonstrated excellent reproducibility of the obtained zeta potential values.

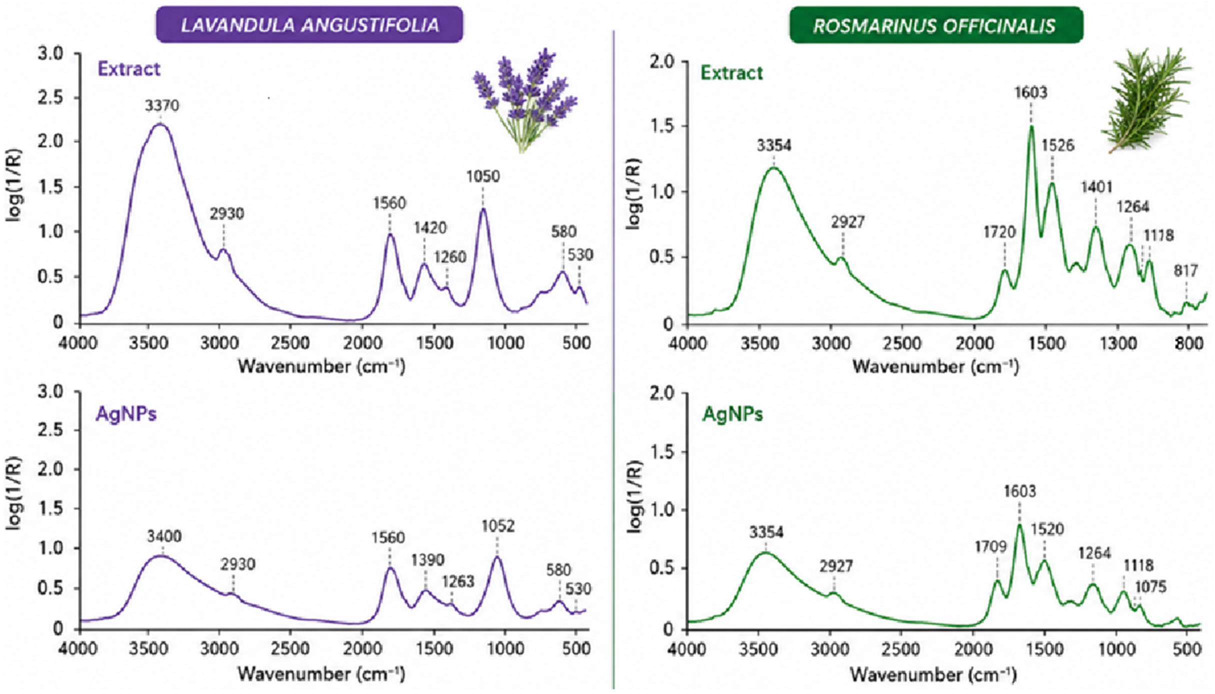

Figure S5. FTIR spectra of *Lavandula angustifolia* and *Salvia rosmarinus* extracts before and after the biosynthesis of silver nanoparticles (AgNPs).

Characteristic absorption bands corresponding to hydroxyl, aliphatic C–H, carbonyl, aromatic, and C–O functional groups are indicated. Slight shifts in band positions and changes in peak intensities after nanoparticle synthesis suggest the involvement of plant-derived phytochemicals in the reduction of Ag^+ ions and the stabilization of the synthesized AgNPs.

FTIR Analysis

FTIR spectroscopy was performed to identify the functional groups present in *Lavandula angustifolia* and *Salvia rosmarinus* extracts and to evaluate their involvement in the biosynthesis of silver nanoparticles (Figure S5).

For Lavandula angustifolia, the FTIR spectrum of the extract exhibited a broad absorption band centered at approximately 3370 cm^{-1}, corresponding to O–H stretching vibrations of hydroxyl groups present in phenolic compounds and alcohols. After nanoparticle synthesis, this band shifted slightly to 3400 cm^{-1} and decreased in intensity, suggesting the participation of hydroxyl-containing phytochemicals in the reduction of Ag^+ ions and their adsorption onto the nanoparticle surface. The absorption band at 2930 cm^{-1}, assigned to aliphatic C–H stretching vibrations, remained almost unchanged after synthesis, indicating that aliphatic chains were only marginally affected during nanoparticle formation.

The band at approximately 1560 cm^{-1}, attributed to C=C stretching vibrations of aromatic rings and/or asymmetric stretching of carboxylate groups, remained at the same position but showed reduced intensity following nanoparticle synthesis, indicating interactions between aromatic phytochemicals and the AgNPs surface. The absorption band at 1420 cm^{-1} shifted to 1390 cm^{-1}, suggesting changes in carboxylate or phenolic functional groups involved in nanoparticle stabilization. Similarly, the band assigned to C–O stretching vibrations shifted slightly from 1260 to 1263 cm^{-1}, while the characteristic peak at 1050 cm^{-1} shifted to 1052 cm^{-1}, indicating the involvement of alcohols, polysaccharides, or

glycosidic compounds during nanoparticle formation. Minor bands observed at approximately 580 and 530 cm^{-1} remained unchanged after synthesis.

For *Salvia rosmarinus*, the FTIR spectrum showed a broad O–H stretching band at approximately 3354 cm^{-1}, characteristic of phenolic compounds. Unlike lavender, no significant shift of this band was observed after nanoparticle synthesis, although a decrease in its intensity suggested its participation in the reduction process. The C–H stretching band at 2927 cm^{-1} also remained unchanged.

More pronounced changes were observed in the fingerprint region. The absorption band at 1720 cm^{-1}, assigned to C=O stretching vibrations of carbonyl-containing compounds, shifted to 1709 cm^{-1}, indicating interactions between carbonyl groups and the nanoparticle surface. The characteristic aromatic band at 1603 cm^{-1} remained unchanged, whereas the adjacent band shifted from 1526 to 1520 cm^{-1}, suggesting structural modifications of aromatic phytochemicals involved in nanoparticle synthesis. Additional changes were observed in the C–O stretching region, where the absorption band at 1118 cm^{-1} shifted to 1075 cm^{-1}, indicating the participation of alcohols, ethers, or polysaccharides in nanoparticle stabilization. The peaks at 1264 cm^{-1} and 817 cm^{-1} remained essentially unchanged after synthesis.

Overall, both plant extracts exhibited slight shifts in characteristic absorption bands together with changes in peak intensities following nanoparticle synthesis. These observations indicate that hydroxyl, carbonyl, aromatic, and C–O-containing phytochemicals participate in both the reduction of silver ions and the stabilization of the synthesized nanoparticles, supporting the proposed mechanism of green synthesis.

Supplementary Material and Methods, Tables S10–S11, and Figures S6–S7.

The supplementary material contains the RP-HPLC-DAD phytochemical profiles of the aqueous extracts of *Lavandula angustifolia* and *Salvia rosmarinus*, together with additional interpretation of observed differences in phenolic composition and extraction-related factors influencing compound recovery.

Supplementary Material and Methods

Aqueous *Lavandula angustifolia* and *Salvia rosmarinus* extracts were filtered through a 0.2 μm nylon membrane prior to analysis. The samples were analysed by reversed-phase high-performance liquid chromatography coupled with diode-array detection (RP-HPLC-DAD) using an Agilent 1260 Infinity Quaternary LC system equipped with a 1260 Infinity diode-array detector (Agilent Technologies, Santa Clara, CA, USA). Chromatographic separation was performed on a Kromasil C18 column (250 × 4.6 mm, 5 μm particle size) maintained at 30 °C. Both aqueous extracts (Lavandula angustifolia and Salvia rosmarinus) were analysed within the same HPLC-DAD analytical sequence under identical chromatographic conditions, allowing direct comparison of their phytochemical profiles. The mobile phase consisted of solvent A (5% acetonitrile containing 0.1% trifluoroacetic acid) and solvent B (100% acetonitrile containing 0.1% trifluoroacetic acid), delivered at a flow rate of 0.8 mL/min. The following gradient elution program was applied: 0 min, 90:10 (A:B); 2 min, 84:16; 10 min, 78:22; 32 min, 70:30; 45 min, 0:100; 58 min, 0:100; and 60 min, 90:10. Compound identification was performed by comparing retention times and UV absorption spectra with those of authentic reference standards. The standards included gallic acid, 4-hydroxybenzoic acid, chlorogenic acid, neochlorogenic acid, cryptochlorogenic acid, *p*-coumaric acid, eriodictyol, and carnosol (Sigma-Aldrich, St. Louis, MO, USA), as well as apigenin, apigenin-7-*O*-glucoside, luteolin, luteolin-7-*O*-glucoside, naringin, naringenin, caffeic acid, catechin, epicatechin, herniarin, and umbelliferone (Extrasynthese, Genay, France). Chromatograms were evaluated at 210, 280, 330, or 350 nm according to the absorption

characteristics of the analysed compounds. Quantification was carried out using external calibration curves, and the results were expressed as concentrations (nmol/mL) in the aqueous extracts. Compounds for which authentic standards were unavailable were tentatively identified based on their UV spectra, retention times, and published literature data. Their concentrations were estimated using calibration curves of the most structurally similar available standards and are expressed as equivalent concentrations (nmol/mL).

Table S10. Phytochemical profile, retention times, molar concentrations, and relative abundances of quantified phenolic compounds in the aqueous extract of *Lavandula angustifolia*.

No.	Compound	Retention time [min.]	Molar concentration [nmol/mL]	Relative abundance [%]
**1**	4-hydroxybenzoic acid	3.0	15.2	0.3
**2**	Gallic acid	4.0	83.7	1.7
**3**	Neochlorogenic acid	6.2	54.6	1.2
**4**	Chlorogenic acid	7.3	83.2	1.8
**5**	Hydroxycinnamic acid derivative	7.6	771.9	16.3
**6**	Cryptochlorogenic acid	8.8	613.6	13.0
**7**	Caffeic acid	9.7	34.7	0.7
**8**	Coumarin derivative	10.4	255.8	5.4
**9**	Rosmarinic acid derivative	12.4	514.2	10.8
**10**	Coumarin derivative	12.9	678.4	14.3
**11**	Luteolin-7-O-glucoside	13.4	20.1	0.4
**12**	6-Hydroxyluteolin-7-O-glycoside	14.0	152.7	3.2
**13**	Umbelliferone	16.1	21.8	0.5
**14**	Naringenin-7-O-glucoside	16.7	17.1	0.4
**15**	Rosmarinic acid	20.1	503.4	10.6
**16**	Apigenin glycoside	22.7	12.2	0.2
**17**	Coumarin derivative	25.7	587.7	12.4
**18**	Naringenin derivative	28.0	77.2	1.6
**19**	Naringenin derivative	31.7	112.0	2.4
**20**	Herniarin	33.1	123.3	2.6
**21**	Naringenin	38.1	3.32	0.1

Note: Relative abundance (%) was calculated as the percentage contribution of each individual compound to the total concentration of all quantified phenolic compounds.

Figure S6. RP-HPLC-DAD chromatographic profile of the aqueous extract of *Lavandula angustifolia* recorded at λ = 280 nm (A) and λ = 330 nm (B).

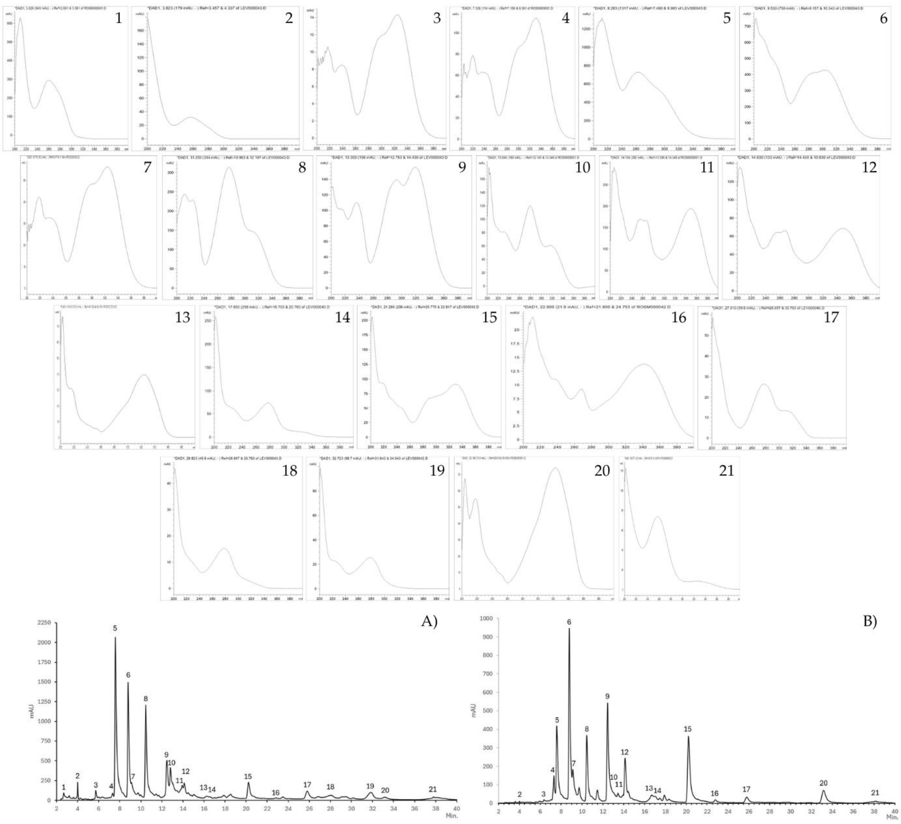

Note: Compound numbers correspond to the compounds listed in Table S10.

Table S11. Phytochemical profile, retention times, molar concentrations, and relative abundances of quantified phenolic compounds in the aqueous extract of *Salvia rosmarinus*.

No.	Compound	Retention time [min.]	Molar concentration [nmol/mL]	Relative abundance [%]
**1**	Gallic acid	4.0	18.4	4.55
**2**	Catechin	5.8	9.7	2.40
**3**	Neochlorogenic acid	6.2	4.17	1.03
**4**	Coumaric acid derivative	7.6	12.8	3.16
**5**	Caffeic acid	9.7	46.7	11.54
**6**	Luteolin-7-O-glucuronide	14.3	14.17	3.50
**7**	Eriodictyol glycoside	16.4	28.1	6.94
**8**	Eriodictyol glycoside	17.0	24.1	5.95
**9**	Apigenin derivative	19.9	15.0	3.71
**10**	Rosmarinic acid	20.3	157.2	38.83
**11**	Apigenin derivative	25.4	9.8	2.42
**12**	Apigenin derivative	26.7	14.7	3.63
**13**	Naringenin	38.3	33.6	8.30
**14**	Cirsimaritin	41.1	8.7	2.15
**15**	Carnosol or carnosol derivative	41.9	1.5	0.37
**16**	Lipophilic caffeic acid derivative	44.9	6.2	1.53

Note: Relative abundance (%) was calculated as the percentage contribution of each individual compound to the total concentration of all quantified phenolic compounds.

Figure S7. RP-HPLC-DAD chromatographic profile of the aqueous extract of *Salvia rosmarinus* recorded at λ = 280 nm (A) and λ = 330 nm (B).

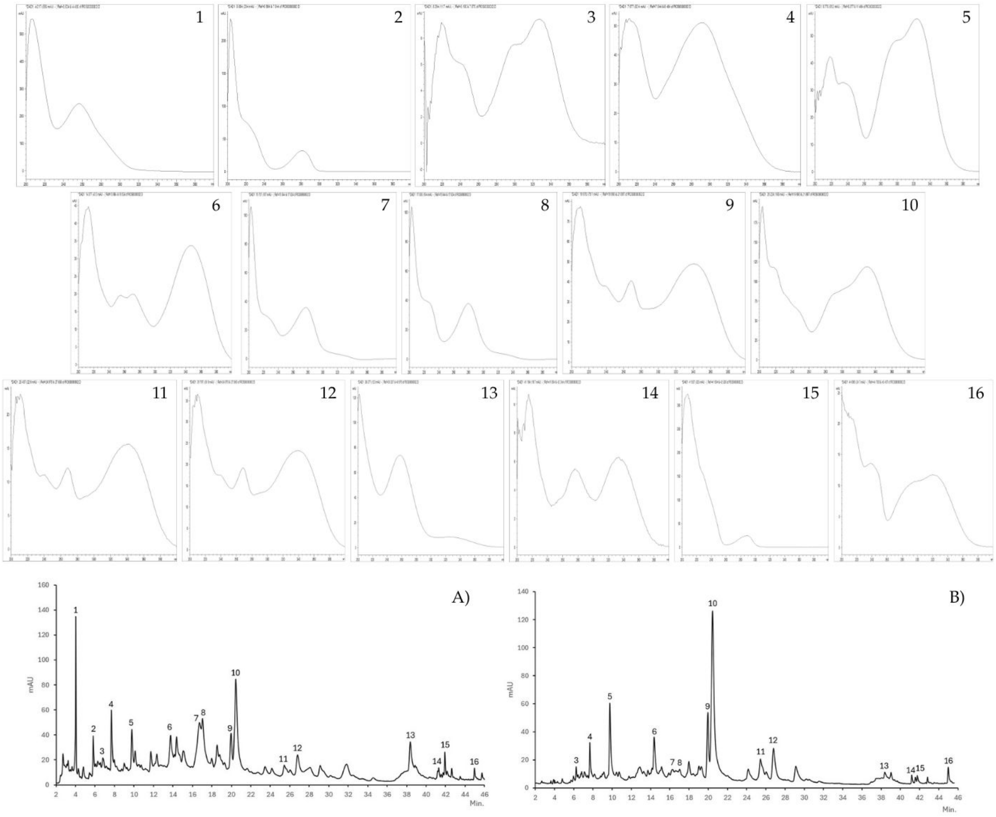

Note: Compound numbers correspond to the compounds listed in Table S11.

Additional discussion on phytochemical profiles of aqueous extracts

The RP-HPLC-DAD analysis revealed considerable differences in the phytochemical profiles of the two aqueous extracts. The total concentration of quantified phenolic compounds was markedly lower in the *Salvia rosmarinus* extract compared with the *Lavandula angustifolia* extract, despite *S. rosmarinus* being generally recognized as a rich source of phenolic compounds, particularly rosmarinic acid and phenolic diterpenes [1,2]. This discrepancy may be related to differences in extraction efficiency. The recovery of phenolic compounds from *S. rosmarinus* is strongly influenced by extraction conditions and solvent selection, with hydroalcoholic or ethanolic extraction approaches commonly used to enhance the extraction of bioactive constituents [2,3]. Therefore, the relatively lower concentrations of detected phenolic compounds and rosmarinic acid in the aqueous rosemary extract observed in the present study may reflect the limited extractability of certain compounds in water rather than their actual abundance in the plant material.

References:

1. Oalđe Pavlović, M.; Milutinović, M.; Alimpić Aradski, A.; Gašić, U.; Mišić, D.; Marin, P.D.; Duletić-Laušević, S. Therapeutic Potential of Salvia rosmarinus: Seasonal and Geographical Variation in Phytochemical Composition, Bioactivity, and Synergistic Effects of Rosmarinic Acid with 5-FU. Plants **2026**, *15*, 1. https://doi.org/10.3390/plants15010001
2. Musolino, V.; Macrì, R.; Cardamone, A.; Tucci, L.; Serra, M.; Lupia, C.; Maurotti, S.; Mare, R.; Nucera, S.; Guarnieri, L.; et al. *Salvia rosmarinus* Spenn. (Lamiaceae) Hydroalcoholic Extract:

Phytochemical Analysis, Antioxidant Activity and In Vitro Evaluation of Fatty Acid Accumulation. *Plants* **2023**, *12*, 3306. https://doi.org/10.3390/plants12183306

3. Dhouibi, N.; Manuguerra, S.; Arena, R.; Messina, C.M.; Santulli, A.; Kacem, S.; Dhaouadi, H.; Mahdhi, A. Impact of the Extraction Method on the Chemical Composition and Antioxidant Potency of *Rosmarinus officinalis* L. Extracts. *Metabolites* **2023**, *13*, 290. https://doi.org/10.3390/metabo13020290
